# Structural and functional distinctions of co-resident microglia and monocyte-derived macrophages after retinal degeneration

**DOI:** 10.1186/s12974-022-02652-2

**Published:** 2022-12-12

**Authors:** Kaitryn E. Ronning, Sarah J. Karlen, Marie E. Burns

**Affiliations:** 1 Center for Neuroscience, University of California, 1544 Newton Court, Davis, CA 95618 USA; 2 Department of Cell Biology and Human Anatomy, University of California, Davis, CA 95616 USA; 3 Department of Ophthalmology & Vision Science, University of California, Davis, CA 95616 USA

**Keywords:** Neuroinflammation, Retina, Photoreceptor, Degeneration, Microglia, Macrophage, Monocyte, Laser damage

## Abstract

**Background:**

Both resident microglia and invading peripheral immune cells can respond to injury and degeneration in the central nervous system. However, after dead and dying neurons have been cleared and homeostasis is re-established, it is unknown whether resident immune cells fully resume normal functions and to what degree the peripheral immune cells take up residence.

**Methods:**

Using flow cytometry, in vivo retinal imaging, immunohistochemistry, and single-cell mRNA sequencing, we assess resident microglia and monocyte-derived macrophages in the retina during and after the loss of photoreceptors in the *Arr1*^*−/−*^ mouse model of inducible degeneration.

**Results:**

We find that photoreceptor loss results in a small, sustained increase in mononuclear phagocytes and, after degeneration wanes, these cells re-establish a spatial mosaic reminiscent of healthy retinas. Transcriptomic analysis revealed the population remained unusually heterogeneous, with several subpopulations expressing gene patterns consistent with mildly activated phenotypes. Roughly a third of “new resident” cells expressed markers traditionally associated with *both* microglial and monocytic lineages, making their etiology ambiguous. Using an inducible Cre-based fluorescent lineage tracing paradigm to confirm the origins of new resident immune cells, we found approximately equal numbers of microglia and monocyte-derived macrophages after degeneration had subsided. In vivo retinal imaging and immunohistochemical analysis showed that both subpopulations remained functionally responsive to sites of local damage, though on average the monocyte-derived cells had less morphological complexity, expressed higher levels of MHCII, and had less migratory activity than the native resident population.

**Conclusions:**

Monocytic cells that infiltrate the retina during degeneration differentiate into monocyte-derived macrophages that can remain in the retina long-term. These monocyte-derived macrophages adopt ramified morphologies and microglia-like gene expression. However, they remain distinguishable in morphology and gene expression from resident microglia and appear to differ functionally, showing less responsiveness to subsequent retinal injuries. These findings support the idea that persistent changes in the local immune population that occur in response to cell loss in aging and progressive retinal diseases may include the establishment of subpopulations of bone marrow-derived cells whose ability to respond to subsequent insults wanes over time.

**Supplementary Information:**

The online version contains supplementary material available at 10.1186/s12974-022-02652-2.

## Background

Neuroinflammation has been implicated in nearly all neurodegenerative disorders. During degeneration, resident macrophages of the central nervous system (CNS), called microglia, undergo changes known as activation, which can involve changes in gene expression and morphology as they retract their highly branched processes to adopt a phagocytic, ameboid state. These changes are the topic of extensive investigations, with the goals of understanding the mechanisms that drive these transformations, determining if they are harmful or helpful to the degenerating tissue, and seeking potential therapeutic targets to modulate their activity. Neurodegeneration can also cause blood–tissue barriers to be breached, with an invasion of immune cells from the periphery joining the microglial response. In the retina, these peripheral cells from the bloodstream are primarily monocytes, which then rapidly differentiate into microglia-like cells within the CNS and can undergo activation themselves. Here, we refer to these monocyte-derived cells as macrophages to distinguish these infiltrating cells from the endogenous microglia residents. Whether these subpopulations of microglia and macrophages serve different functions or have different fates in the CNS is not known.

One CNS compartment commonly used to study microglia and macrophages during degeneration is the retina [9]. Many inherited retinal degenerations like retinitis pigmentosa involve the loss of rod and cone photoreceptors. As photoreceptors die, the remaining retina remodels into a relatively stable homeostatic state that is considered ideal for optogenetic gene therapy or implants to restore light sensitivity to the remaining circuitry [25]. The status of the immune population in the retina during this period is of paramount importance for understanding the response to subsequent insults and for rational therapeutic design, particularly those utilizing virus-mediated gene transfer.

Previously, we found that microglia activate and pro-inflammatory monocytic cells invade the retina during inducible photoreceptor degeneration (*Arr1*^*−/−*^ mouse; [10, 12, 24]). However, the fates of these cells after photoreceptor loss were unknown. Here, using a combination of single-cell mRNA sequencing, in vivo retinal imaging, and in vitro validations, we investigate the state of retinal immune cells after photoreceptor degeneration has waned and the retina has returned to homeostasis. The expression patterns of key genes allowed us to identify the relative activation states of subpopulations with distinct lineages and to probe the responsiveness of these subpopulations to a second retinal injury.

## Methods

### Animals

Mice were cared for and handled in accordance with the National Institutes of Health guidelines and under protocols approved by the UC Davis Institutional Animal Care and Use Committee. All mice were born and maintained in constant darkness before exposure to constant light (approximately 200 lx) and were 2–5 months old when used for experiments, except for lineage mice which were treated with tamoxifen at 6–12 weeks and used for experiments at 4–9 months of age (after fluorescent cell markers were established). All mouse strains were tested to confirm the absence of the *rd8* mutation. Although there are some reports of potential sex differences in microglial function [36], there were no observable sex differences in immunohistochemistry, flow cytometry, or in vivo imaging experiments, so both male and female mice were used for these experiments. Only female mice were used for single-cell mRNA sequencing to avoid potential confounding sex-specific differences in gene expression.

*Arr1*^*−/−*^ mice [2, 38] are a convenient, light-inducible model of photoreceptor degeneration. C57BL/6J mice (strain 000664), originally obtained from The Jackson Laboratory and then bred in constant dark conditions, were used as controls for some experiments. *Cx3cr1-GFP* mice (strain 005582, aka B6.129P2(Cg)-*Cx3cr1*^*tm1Litt*^/J)[8] were obtained from The Jackson Laboratory and crossed with the *Arr1*^*−/−*^ strain to obtain *Arr1*^*−/−*^*Cx3cr1*^+*/GFP*^ mice. Littermate *Arr1*^+*/*+^*Cx3cr1*^+*/GFP*^ mice were used as controls. For lineage tracing experiments, Ai9 (strain 007909, aka B6.Cg-*Gt(ROSA)26Sor*^*tm9(CAG−tdTomato)Hze*^/J)[16] and *Cx3cr1-YFP-CreER* (strain 021160, aka B6.129P2(Cg)-*Cx3cr1*^*tm2.1(cre/ERT2)Litt*^/WganJ)[13] mice were obtained from The Jackson Laboratory and crossed with the *Arr1*^*−/−*^ strain to obtain *Arr1*^+*/*+ *or −/−*^* Ai9*^*KI/KI*^* Cx3cr1*^+*/YFP−CreER*^ mice for lineage tracing experiments.

### In vivo retinal imaging and focal light damage

A custom-built dual scanning laser ophthalmoscopy (SLO) and optical coherence tomography (OCT) system was used to simultaneously collect structural and fluorescent information from the retina non-invasively [40]. For imaging, mice were anesthetized with 2–2.5% isoflurane and kept on a 37 °C heating pad. A micropositioner (Biptigen, Morrisville, NC) was used for fine rotational and translational movements of the pad to position the mouse. Pupils were dilated with tropicamide and phenylephrine (Akorn), and then hypromellose gel (GenTeal Tears Severe, Alcon) was applied to the corneal surface. This gel both prevented the cornea from drying during imaging and maintained the refractive index between the cornea and contact lens (Unicon Corporation). SLO imaging was performed with a 488-nm laser, approximately 100–130 μW at the pupil, to excite GFP. Wide-field images were collected over 51° of visual angle, and increased digital sampling was used to “zoom” in on areas of interest. SLO images were processed and analyzed in FIJI [26], including registering images with the MultiStackReg plugin (a modified version of the TurboReg plugin [34] written by Brad Busse), averaging frames, and pseudo-coloring images for presentation.

OCT imaging was performed with a superluminescent diode centered at 860 nm with a 132-nm bandwidth (Broadlighter T-860-HP, Superlum), approximately 600 μW at the pupil. The FD-OCT data were processed using standard methods to transform the back-scattered light into intensity by depth information and then generate a series of OCT B-scans. As in our previous study [19], a custom Python script was used to flatten the B-scans and then register those flattened B-scans using a strip-registration algorithm to generate complete flattened OCT volumes. As previously described [19], focal light damage was performed by maintaining the OCT laser at a single location at full power (7–8 mW) for 2 min.

### Intraperitoneal injections

Intraperitoneal (IP) injections were used to deliver 5-ethynyl-2’-deoxyuridine (EdU) and tamoxifen for proliferation and lineage tracing experiments, respectively. For the proliferation experiments, EdU was dissolved in sterile 1 × phosphate buffered saline (PBS) at a concentration of 10 mg/mL and delivered at a dose of 100 mg/kg (or 10 ml/kg of the EdU solution). EdU was administered to dark-reared *Arr1*^*−/−*^ and C57BL/6J mice, approximately 2–4 months of age, at 48 and 72 h after the onset of light exposure. These are the time points that previous data [10] indicated microglia and macrophages are mitotically active. For the lineage tracing experiments, tamoxifen was dissolved in corn oil at a concentration of 20 mg/ml. *Arr1*^+*/*+ *or −/−*^* Ai9*^*KI/KI*^* Cx3cr1*^+*/CreER−YFP*^ mice received two IP injections of tamoxifen at a dosage of 75 mg /kg (or 3.67 ml/kg of the corn oil solution), separated by 1 day as previously described [20]. These IP injections were performed when the mice were approximately 6–12 weeks of age, and dark lighting conditions were maintained during injections. Further experiments and light exposure did not proceed until at least 60 days after the tamoxifen injections to allow all the monocytes to turn over, leaving only long-lived resident macrophages dual-labeled.

### Immunohistochemistry

Immunohistochemistry was performed as previously described [24]. Immediately after enucleation eyes were submerged in 4% paraformaldehyde at room temperature. After approximately 5 min of fixation, anterior segments and lenses were removed, the resulting eyecups were fixed for an additional 20–25 min at room temperature, and then fixed eyecups were transferred to vials of PBS at 4 °C. To remove the retinal flat mount from the eyecup, several curvature-releasing cuts were made with small dissection scissors, and then small forceps were used to gently peel the retina from the rest of the eyecup. For antibody staining, retinas were incubated in 1% Triton X-100 in PBS overnight at 4 °C, blocked with normal serum for 2 h at 37 °C, incubated in primary antibodies in PBS with 0.5% bovine serum albumin and 0.5% Triton X-100 (PBT) overnight at 4 °C, rinsed 3 times in PBT for 10 min at room temperature, incubated in secondary antibodies for 1.5–2 h at 37 °C, rinsed in PBT again at room temperature, and then mounted on glass slides using ProLong Diamond Antifade Mountant (Invitrogen). For proliferation (EdU) experiments retinal flat mounts were permeabilized in 1% Triton X-100 for 45 min at room temperature, followed by using the Click-iT EdU Alexafluor 488 Imaging Assay (C10337, Invitrogen) to label EdU according to manufacturer instructions. Next retinal flatmounts were blocked with normal serum and then incubated with pre-conjugated antibodies, both for approximately 2 h at 37 °C. Finally, these flatmounts were rinsed 2 times in PBT for 10 min, once in PBS for 10 min, and mounted as above. See Table 1 for a full list of antibodies and labeling reagents used for immunohistochemistry. All retinal flatmounts were imaged using a Nikon A1 confocal microscope.

**Table 1:** Antibodies and labels used for immunohistochemistry and flow cytometry

Reagent	Manufacturer	Catalog number	RRID	Application	Dilution
Goat anti-GFP antibody	Rockland	600-101-215	AB_218182	IHC	1:200
Alexa Fluor 647 anti-CD11b antibody	Biolegend	101218	AB_389327	IHC	1:300
Alexa Fluor 594 anti-I-A/I-E (MHCII) antibody	Biolegend	107650	AB_2566438	IHC	1:200
Alexa Fluor 647 anti-I-A/I-E (MHCII) antibody	Biolegend	107617	AB_493526	IHC	1:200
Rabbit anti-RFP antibody	Rockland	600-401-379	AB_2209751	IHC	1:750
Click-iT EdU kit for imaging, Alexa Fluor 488	Invitrogen	C10337	None	IHC	NA
Zombie NIR Viability Dye	Biolegend	423105	None	FC	1:500
APC anti-CD45 antibody	Biolegend	103112	AB_312977	FC	1:100
PE anti-CD45 antibody	Biolegend	103106	AB_312971	FC	1:100
BV605 anti-CD11b antibody	Biolegend	101257	AB_2565431	FC	1:100
BV711 anti-I-A/I-E antibody	Biolegend	107643	AB_2565976	FC	1:100
Alexa Fluor 488 anti-Ly6C antibody	Biolegend	128022	AB_10639728	FC	1:100
BV421 anti-CX3CR1 antibody	Biolegend	149023	AB_2565706	FC	1:100

*IHC* immunohistochemistry, *FC* flow cytometry

Maximum intensity projection images were created using NIS-Elements software (Nikon). ImageJ [26] was used to create the pseudocolored-overlay images shown in Figs. 4A and 6B; method depicted in Additional file 3: Fig. S3. Single-channel images were first thresholded using Color Threshold, then converted to a binary mask and overlayed using the Image Calculator set to Average. For YFP images with large amounts of background, speckle noise was removed using Remove Outliers (1–3 pixels) prior to processing. Grayscale overlay images from ImageJ were taken to Photoshop (Adobe), where cells were manually pseudocolored peach if dual-positive or blue if single-positive. To quantify the response to secondary focal damage in Fig. 6C, we counted the number of pixels of each color within the injury area. For the Sholl analysis in Fig. 5, maximum intensity projections were exported from NIS-Elements and binary images of individual cells were generated in ImageJ. An ImageJ plugin was used to perform the Sholl analysis [5], with a soma radius of 20 pixels and using 10 pixel steps. The number of primary branches, required for the calculation of the ramification index, was identified manually. For Fig. 5, cells with detectable levels of MHCII immunostaining were categorized as MHCII^high^ while undetectable levels were categorized as MHCII^low^ since we could not be sure based solely on IHC that cells were MHCII negative.

### Flow cytometry and fluorescence activated cell sorting

Retinal samples were prepared similarly to a previously described protocol [10]. Approximately 5 min before euthanasia mice received a tail vein injection of anti-mouse CD45 antibody conjugated to either PE or APC, to differentiate cells within the vasculature from those in the retinal parenchyma [21]. Mice were then euthanized, enucleated, and then retinas isolated. Isolated retinas were placed in 1 mL of digestion buffer, consisting of Hibernate medium (HBSS, 10-547F, Lonza) supplemented with 5% fetal bovine serum (FBS), 10 mM HEPES, 0.7 mg/mL calcium chloride, 1.5 mg/mL collagenase A (Roche), and 0.1 mg/mL DNase I (Roche). Tissue was incubated at 37 °C for 15–20 min, with mild trituration for all samples ultimately used for single-cell sequencing, or gently disrupted with a MACS Octo Dissociator (Miltenyi Biotec) for all samples used for flow cytometry. After digestion, the cell suspension was filtered through a 70-µm cell strainer (Genesee Scientific) and resuspended in PBS. Cells were then stained for viability (Zombie NIR Fixable Viability Kit, 423106, BioLegend), blocked for 5 min with Fc block 1 µl/sample (eBioscience) supplemented with 5% each normal rat and mouse sera, and then incubated with antibodies diluted in Brilliant Stain Buffer (BD Biosciences) for 15–30 min at room temperature. Table 1 includes a complete list of the antibodies and cell stains used for these experiments. After staining, cell suspensions were washed in 0.5% bovine serum albumin (BSA) with 1:50 EDTA, and ultimately resuspended in either the same buffer for cell sorting experiments or Cell Staining Buffer (BioLegend) for flow cytometry experiments. Additionally, flow cytometry samples were then fixed by adding 0.5% paraformaldehyde to the final cell suspensions. Control beads were prepared using the AbC Total Antibody Compensation Bead Kit (Invitrogen) and ArC Amine Reactive Compensation Bead Kit (Invitrogen) following manufacturer guidelines. Blood samples taken and processed in parallel as before [10] were used to set the CD45-IV gate for each experiment.

All flow cytometry and cell sorting was performed at the UCD Flow Cytometry Shared Resource Laboratory. For the time course flow cytometry experiment, samples were run on an LSR-II Cytometer (Becton Dickinson). For quantifying cells after lineage tracing, samples were run on a Cytoflex Cytometer (Beckman Coulter). Cell sorting prior to single-cell mRNA sequencing was performed on an Astrios EQ Cell Sorter (Beckman Coulter). FlowJO software (Tree Star) was used for analyses.

### Statistical analysis

**Fig. 1:**
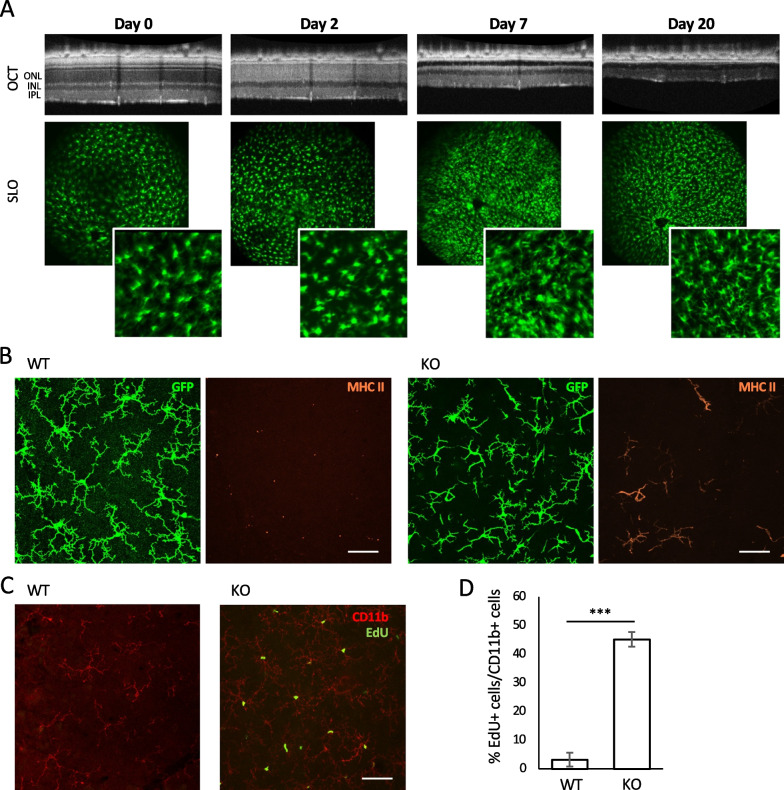
Ramified macrophages re-tile the retina after widespread photoreceptor degeneration. **A** Representative in vivo retinal imaging to track retinal thinning (OCT) and macrophages (SLO) in an *Arr1*^*−/−*^*Cx3cr1*^+*/GFP*^ mouse. **B** Maximum intensity projections through ~ 10 μm the inner plexiform layer of *Arr1*^+*/*+^*Cx3cr1*^+*/GFP*^ (WT) and *Arr1*^*−/−*^*Cx3cr1*^+*/GFP*^ (KO) retinas after 20 days of light exposure. Retinal flatmounts were labeled for GFP (green) and MHCII (orange) using IHC. **C** Maximum intensity projections through the inner plexiform layer of C57BL/6J (WT) and *Arr1*^*−/−*^ (KO) after 20 days of light exposure, pulsed with EdU at 2 and 3 days of light exposure. Macrophages are labeled with CD11b (red) and EdU^+^ nuclei are indicated in green. **D** Quantification of **C**, showing the percentage of EdU^+^ CD11b^+^ cells out of all CD11b^+^ cells in the inner plexiform layers of KO and WT retinas, pulsed with EdU at 2 and 3 days, probed at 20 days of light exposure. Percentages displayed as mean ± SE; *n* = at least 7 images per genotype; ***p < 0.001. ONL = outer nuclear layer; INL = inner nuclear layer; IPL = inner plexiform layer. Scale bars indicate 50 μm

**Fig. 2:**
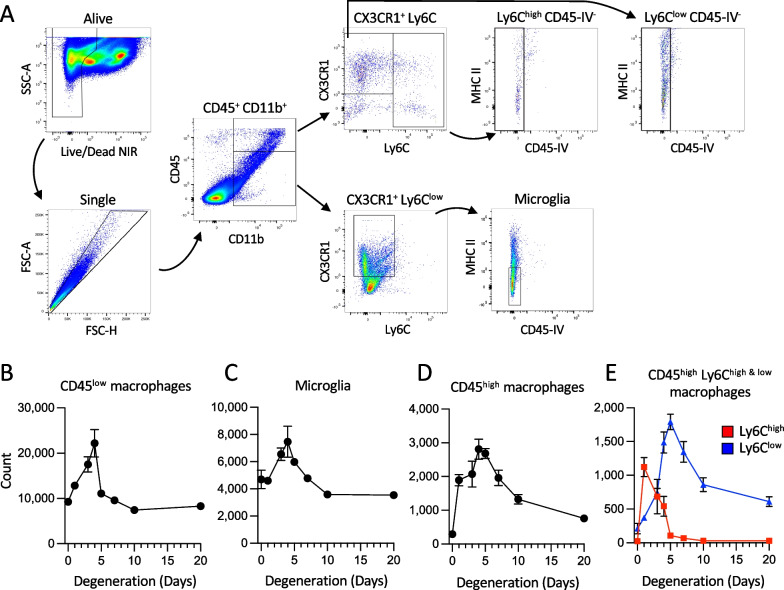
Persistent increase in CD45^high^ macrophages after degeneration. **A** Flow cytometry gating strategy. CD45-IV (intravenous anti-CD45 antibody) was used to exclude immune cells circulating in the retinal vasculature at tissue collection. **B** Quantification of CD45^low^ macrophages, which transiently increase in number during degeneration and then return to baseline. **C** Quantification of CD45^low^ CD11b^+^ Cx3cr1^+^ Ly6C^low^ MHCII^low^ CD45-IV^−^ microglia, which also transiently increase in number during degeneration and then return to baseline. **D** Quantification of CD45^high^ macrophages, which transiently increase in number then decrease, but do not fully return to baseline numbers. **E** CD45^high^ Ly6C^high^ cells (red) increased rapidly following light onset, and then returned to baseline by Day 5. In contrast, CD45^high^ Ly6C^low^ cells (blue) increased in number more slowly and did not fully return to baseline. *n* = 2–6 retinas per timepoint from the following numbers of mice: 0 days (*n* = 2), 1 day (*n* = 3), 3 days (*n* = 3) 4 days (*n* = 2), 5 days (*n* = 2), 7 days (*n* = 2), 10 days (*n* = 2), and 20 days (*n* = 2). Mean ± SE where *n* > 3 retinas

In all figures, data are represented as mean ± standard error and significance levels are indicated as follows: *p < 0.05, **p < 0.01, ***p < 0.001. All statistical analyses were performed using R version 4.0.0 or higher (R Core Team, n.d.). Two group comparisons (as in Figs. 1D and 5D) were performed using t-tests. Multiple group comparisons of immunohistochemistry and flow cytometry data (as in Figs. 2F–H and 5B–E) were performed using a one-way ANOVA followed by a Tukey’s honest significant differences test. Multiple group comparisons over time (as in Figs. 4C and 6C) were performed using a two-way ANOVA followed by a Tukey’s honest significant differences test. When any flow cytometry data were highly positively skewed, the data were first logarithmically transformed to improve the normality of the distribution and then statistics were run on the transformed data. Transformed data were never directly compared to non-transformed data, and only non-transformed data are shown.

### Single-cell sequencing and bioinformatics

Single-cell mRNA sequencing was performed as previously described [24]. Briefly, retinal samples were enriched for immune cells by FAC-sorting single, alive, CD45^+^CD11b^+^ cells, as described above. Cells were sorted into a solution of Dulbecco’s modified Eagle medium (DMEM; Thermo Fisher) supplemented with 10% FBS, and then immediately used to create the expression libraries. These libraries were generated using the 10X Genomics system (Chromium) at the UC Davis DNA Technologies and Expression Core, following manufacturer recommendations. The resulting cDNA libraries were sequenced on a NovaSeq (Illumina), again following manufacturer recommendations.

After sequencing, initial processing including barcode processing, transcript alignment, and generation of gene–barcode matrices was performed using Cell Ranger (Chromium) at the UC Davis Bioinformatics Core. All further processing and analysis was performed in R (R Core Team, n.d.), primarily using the Seurat R package [1]. Following the recommended Seurat workflow, cells were filtered for quality control, and then normalized for UMI and mitochondrial counts. After principal components analysis (PCA), we performed tSNE dimensional reduction and used marker gene expression to identify general cell types. Despite the FAC sorting, there was a small amount of contamination of non-immune cells, including photoreceptors. These cells were excluded, and then PCA, tSNE reduction, and clustering were performed on the remaining myeloid cells. Transcriptionally distinct clusters of cells were identified using Seurat’s graph-based clustering algorithm, and then cell types were assigned based on the relative expression of standard marker genes. Differentially expressed genes were identified with the FindMarkers function in Seurat using the Wilcoxon rank sum test, and only genes with an adjusted *p*-value below 0.05 were considered significantly differentially expressed. The cross-correlational analysis function in Seurat was used to combine this dataset with the previously published data from [24]. Gene enrichment analysis was performed using the Gene Ontology Panther tool [18].

## Results

### Ramified macrophages re-tile the retina after photoreceptor degeneration

During photoreceptor degeneration, microglia adopt an ameboid morphology and migrate to the outer nuclear layer [30, 39]. In the *Arr1*^*−/−*^ mouse model of light-induced photoreceptor degeneration, an influx of monocytes from the periphery and the proliferation of both the resident microglia and invading monocyte-derived macrophages contribute to a dramatic increase in the number of macrophages in the degenerating retina [9, 10]. To understand how this immune response resolves, we began by tracking Cx3CR1-GFP^+^ cells in the retina over time in vivo using *Cx3cr1*^+*/GFP*^ mice, which express GFP in both microglia and monocyte-derived cells. Dark-reared *Arr1*^*−/−*^*Cx3cr1*^+*/GFP*^ and control *Arr1*^+*/*+^*Cx3cr1*^+*/GFP*^ mice were exposed to light, then periodically imaged using scanning laser ophthalmoscopy (SLO) to track the distribution and morphology of GFP^+^ cells, and optical coherence tomography (OCT) to follow the concurrent degeneration (Fig. 1A). As previously reported [10, 24], we observed a dramatic increase in the number of GFP^+^ cells in *Arr1*^*−/−*^ retinas during photoreceptor degeneration (Day 2), and those cells were mostly ameboid in morphology. By 7 days photoreceptor loss was largely complete as revealed by the disappearance of the outer nuclear layer in OCT. At later times (Day 20), there appeared to be a fewer GFP^+^ cells compared to Day 7 and the morphology of many cells were again noticeably more complex and ramified (Fig. 1A).

To visualize the cell morphology at higher resolution, we amplified the GFP signal using immunohistochemistry (IHC) and performed confocal imaging (Fig. 1B, green). In retinal flatmounts, the GFP^+^ cells were ramified with complex secondary and tertiary processes in both WT and KO retinas. This is in stark contrast with the ameboid shape that these cells adopt during photoreceptor cell loss (for example, see [24]). However, the cells in the KO retinas had noticeable differences from the WT microglia mosaic, including more variable degrees of morphological complexity. Additionally, some cells expressed MHCII proteins (Fig. 1B, orange), which are expressed in monocyte-derived macrophages and sometimes activated microglia. Virtually no such MHCII^+^ cells were detectable in WT retinas processed in parallel.

Previously, we found that subpopulations of both microglia and monocyte-derived macrophages in the retina proliferate during the first few days of photoreceptor degeneration [24]. To determine if dividing cells survive and contribute to the retinal macrophage population after degeneration is complete, we pulsed C57BL/6J and *Arr1*^*−/−*^ mice with EdU at days 2 and 3 of light exposure, when cell proliferation has been previously detected in the *Arr1*^*−/−*^ model. Subsequently, on Day 20 eyes were collected and flat mounted retinas were stained for CD11b and EdU and visualized on a confocal microscope. Many EdU^+^ CD11b^+^ cells were observed in all layers of degenerated *Arr1*^*−/−*^ retinas, and only rarely observed in control (C57BL/6J) retinas (Fig. 1C). Cell counting from the histological images revealed approximately 45% of the CD11b^+^ cells were also EdU^+^ in the 20-day retinas (Fig. 1D). These results indicate that just under half of the immune cells in this newly homeostatic state were born during the intense period of photoreceptor degeneration roughly 2 weeks prior.

### Sustained increase in retinal immune cells after degeneration

Although SLO imaging revealed fewer GFP^+^ cells at 20 days than at 7 days, there were still qualitatively more GFP^+^ cells in the retina at this late time compared to healthy controls (Fig. 1A, compare Day 0 to Day 20). To further quantify myeloid cells before, during, and after photoreceptor loss we used flow cytometry. We began by using general myeloid cell gating, identifying alive, single, CD11b^+^, CD45^+^ cells, and then further divided these cells by their degree of CD45 expression (see Fig. 2A for gating). Cells with relatively low CD45 expression initially increased in number, peaking at approximately 4 days of light exposure, and then gradually returned to baseline numbers (Fig. 2B). Low CD45 expression is often a feature of tissue-resident macrophages, so we further gated these cells using a standard microglia paradigm including Cx3cr1^+^, Ly6c^low^, and MHCII^low^ expression. These microglia followed the same temporal pattern as the larger CD45^low^ gate, initially increasing and then returning to baseline cell numbers (Fig. 2C). Myeloid cells with higher CD45 expression also increased in number during the first few days of degeneration and then later decreased (Fig. 2D). Because high CD45 expression is typically a feature of peripherally derived cells, we further differentiated these CD45^high^ cells by their relative Ly6C expression, which is typically high in newly born monocytes. Cells that were Ly6C^high^ increased in number early in degeneration, peaked during the first day of light exposure, and then decreased to baseline numbers within approximately one week (Fig. 2E, red). In contrast, the Ly6C^low^ population peaked a few days later than the Ly6C^high^, and never fully returned to baseline cell numbers (Fig. 2E, blue). These results suggest that the persistent increase in Cx3CR1^+^ cells that we observed after degeneration had waned (Fig. 1A) was due in part to continued presence of monocyte-derived macrophages. Thus, unlike the largely homogenous retinal macrophage population in healthy retinas, the retinal immune cell population remains heterogeneous long after the loss of photoreceptors.

### Persistent transcriptional heterogeneity of retinal macrophages after loss of photoreceptors

**Fig. 3:**
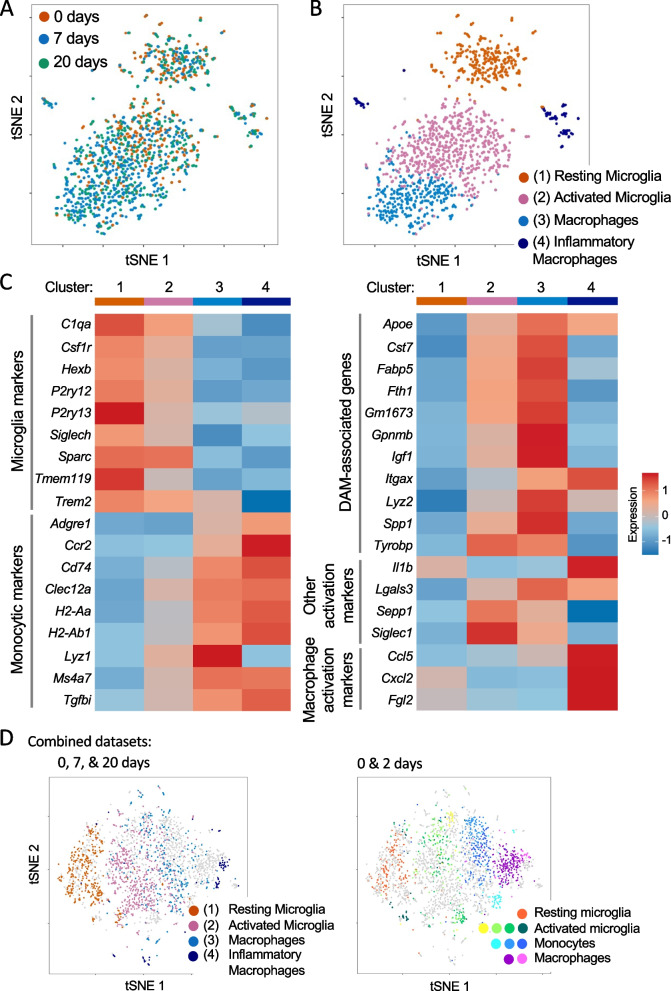
Single-cell mRNA sequencing reveals distinct populations of resident immune cells after degeneration. **A** tSNE plot with cells color-coded by time point (*n* = 8 retinas (4 mice) per timepoint). **B** tSNE plot with the same cells color-coded by cluster. **C** Heatmaps showing the average gene expression in each cluster of genes associated with microglial and monocytic cell lineages (left), and genes associated with microglial and monocyte-derived macrophage activation (right). Disease-associated microglia (DAM) genes were identified previously [11, 31], as were the macrophage activation markers [28]. **D** tSNE plot from this dataset combined with a previously published dataset of retinal macrophages before (0 days) and relatively early (2 days) during photoreceptor loss [24]. On the left, cells are color-coded using the same cluster identities as in **B**, with grey indicating cells from the previously published dataset. On the right, cells from the current dataset are indicated in grey, while cells from the previous dataset are color-coded according to clusters identified in [24]. For simplicity, the cluster naming has been collapsed across general cell types

To investigate the degree of heterogeneity of the new resident population, we performed single-cell sequencing on FACS-enriched retinal immune cells before, immediately following, and well past photoreceptor loss in *Arr1*^*−/−*^ retinas (0, 7, and 20 days, respectively). At all three time points, retinal CD45^+^ cells showed broad transcriptional similarities (Fig. 3A), suggesting that by 1 week many cells had returned to a “resting” state similar to microglia in a healthy retina. To further probe for heterogeneity, we identified transcriptionally distinct clusters of cells using Seurat’s graph-based clustering algorithm (Fig. 3B), and then evaluated expression of known marker genes (see Fig. 3C for examples) to determine the putative identities of these clusters. This analysis identified several distinct subpopulations, including resting microglia, mildly activated microglia, and a small number of inflammatory macrophages (Fig. 3B). Interestingly, the 7 and 20-day samples contained a unique cluster, Cluster #3, of hybrid-like cells expressing some genes traditionally associated with only microglia and specific disease-associated activated microglia [11, 31], such as *Trem2*, *Apoe*, *Fabp5*, and *Spp1*, as well as genes traditionally associated only with cells of a monocytic lineage, such as *Adgre1*, *Cd74*, and *H2-Aa* (Fig. 3C).

When we probed the expression of common pro- and anti-inflammatory related genes, the only cluster with significant expression of either was the cluster containing a very small number of inflammatory macrophages (Cluster #4). All other clusters lacked strong expression of both pro- and anti-inflammatory markers. This suggests that, although Clusters #2 and #3 are distinct from traditional “resting” microglia, these macrophages are not strongly activated. This is especially apparent when examining canonical resting microglia markers, like *Hexb, P2ry12,* and *Siglech*, and activated microglial markers, such as *Lyz2, Sepp1,* and *Apoe* (Fig. 3C). Specifically, in Clusters #2 and #3 resting microglia marker expression was slightly lower on average and more variable, while the activated markers were somewhat higher than in Cluster #1.

The similarities between the mildly activated microglia in Cluster #2 and the putative monocyte-derived macrophages in Cluster #3 prompted us to look for differentially expressed gene programs between these groups. We identified the top 35 most highly differentially expressed genes in the monocytic cells compared to the microglia and performed gene enrichment analysis (Additional file 1: Fig. S1A). The most enriched gene programs included macrophage activation, regulation of cell migration, antigen processing and presentation, and response to interferon-gamma. These enriched gene sets align with a monocyte-derived macrophage phenotype. We also performed gene enrichment analysis on the top 400 most highly expressed genes in the monocytic cluster, which yielded similar results (Additional file 1: Fig. S1B).

Finally, we combined this dataset with a previously published dataset from this model at an earlier timepoint during active degeneration (*t* = 2 days; [24]). A tSNE plot was used to graphically compare the similarities between clusters identified in these two experiments (Fig. 3D). The control (0 days) resting microglia clusters from these two datasets largely overlapped. Especially notable in this plot is that Cluster #3 is located between the monocytic cells from the earlier timepoints, and the activated microglia from both datasets. This is in agreement with the seemingly hybrid phenotype of both intermediate microglial and monocytic gene expression in this cluster.

### Monocyte-derived macrophages take up residence alongside resident microglia

Given the expression of both microglial and monocyte-derived macrophage genes in the hybrid-like cells of Cluster #3, we hypothesized that many monocyte-derived cells were adopting a microglia-like phenotype and remaining in the retina long-term. To test this hypothesis, we used an inducible fate-mapping paradigm to track the lineages of retinal immune cells after degeneration. In this lineage tracing paradigm, all *Cx3cr1*-expressing myeloid cells, including microglia and monocyte-derived cells, express YFP and a tamoxifen-inducible Cre (a Cre-ERT2 fusion protein, referred to here as CreER). Upon tamoxifen administration, Cre-mediated recombination of the Ai9 reporter results in tdTomato expression in all *Cx3cr1*-expressing cells. Then, 60 + days after tamoxifen, by which time all the monocytes have turned over, long-lived resident macrophages, including bona fide microglia, are YFP^+^ and tdTomato^+^, whereas the newly born cells from a monocytic lineage are only YFP^+^ [20].

**Fig. 4:**
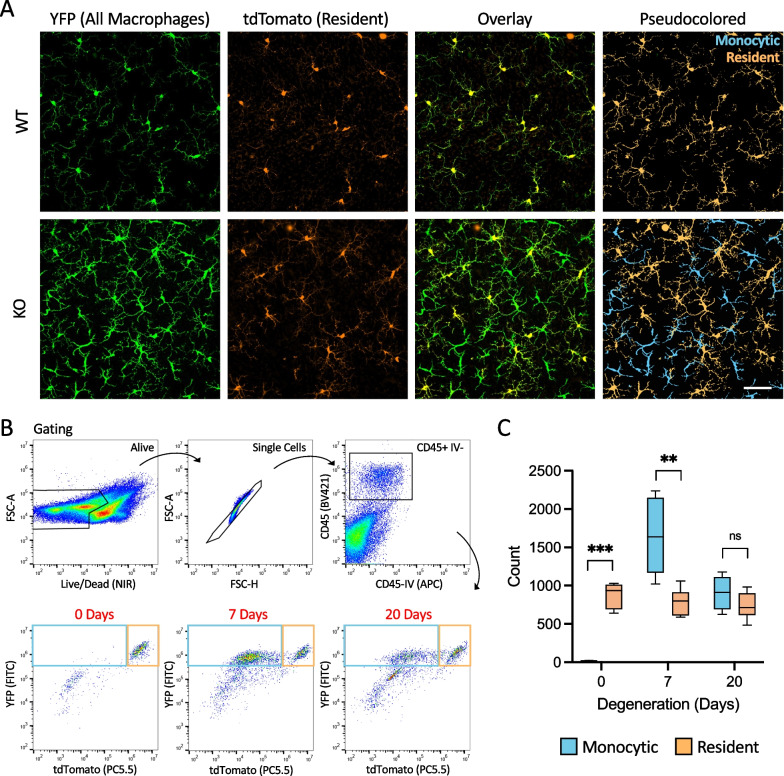
Monocyte-derived macrophages join resident microglia in the resident immune mosaic following acute photoreceptor degeneration. **A** Maximum intensity projections through the inner plexiform layers of WT and *Arr1*^*−/−*^ (KO) retinas after 20 days of light exposure utilizing an inducible fluorescent lineage tracing paradigm (*Arr1*^+*/*+ *or −/−*^* Ai9*^*KI/KI*^* Cx3cr1*^+*/YFP−CreER*^ post-tamoxifen). Resident macrophages express both YFP and tdTomato, whereas monocyte-derived macrophages express only YFP. In the pseudocolored images, YFP^+^tdTomato^+^ (resident) cells have been pseudocolored peach, and YFP^+^tdTomato^−^ (monocytic) cells have been pseudocolored blue. Scale bar indicates 50 μm. **B** Gating for flow cytometry to quantify resident and peripherally derived macrophages. An intravenous CD45 antibody (CD45-IV) was used to exclude immune cells in the vasculature that may have been captured during tissue dissection. **C** Quantification of resident (YFP^+^tdTomato^+^) and peripherally derived (YFP^+^tdTomato^−^) macrophages using flow cytometry before (0 days), immediately after (7 days), and well after (20 days) photoreceptor loss (mean ± SE). **p < 0.01, ***p < 0.001; *n* = 6 retinas (3 mice) per time point

To examine the lineage of the cells remaining in the retina after photoreceptor degeneration had ended, we administered the tamoxifen prior to the onset of any degeneration, in both WT and *Arr1*^*−/−*^ dark-reared animals. After nine weeks, well after the required 60-day period for monocyte turnover, we exposed both strains to light (~ 200 lux) to initiate degeneration. Following degeneration (20 days), we examined both control and *Arr1*^*−/−*^ retinas for dual- and single- labeled macrophages (Fig. 4A). In control retinas (WT, *Arr1*^+*/*+^*Ai9*^*KI/KI*^* Cx3cr1*^+*/YFP−CreER*^ post-tamoxifen), all of the retinal microglia were dual-labeled, as expected. In contrast, in the degenerated retinas (KO, *Arr1*^*−/−*^*Ai9*^*KI/KI*^* Cx3cr1*^+*/YFP−CreER*^ post-tamoxifen), single-labeled cells were abundant. These cells were distributed across all retinal layers and appeared intermixed with dual-labeled cells within the macrophage mosaic. These results demonstrate that following degeneration, infiltrated monocyte-derived cells take up long-term residence in the retina. Furthermore, these single-labeled cells had ramified morphologies, supporting the idea that these monocyte-derived macrophages adopted a microglia-like phenotype (Fig. 4A).

To quantify the relative numbers of both resident (CD45^+^YFP^+^tdTomato^+^) and monocyte-derived (CD45^+^YFP^+^tdTomato^−^) myeloid cells, we performed flow cytometry before and after photoreceptor loss (for gating, see Fig. 4B). We found that in the healthy retina, virtually all myeloid cells were resident cells (YFP^+^tdTomato^+^) (Fig. 4C, 0-day time point). However, by 7 days the dominant myeloid cell type was peripherally derived (YFP^+^tdTomato^−^), with the number of resident cells unchanged from the initial baseline (Fig. 4C, 7-day timepoint). By 20 days of light exposure, the populations of resident and peripherally derived macrophages were equivalent in size (Fig. 4C, 20-day timepoint). These results held even when the counts were normalized to the total number of recorded cells or the number of CD45^+^ cells (Additional file 2: Fig. S2).

**Fig. 5:**
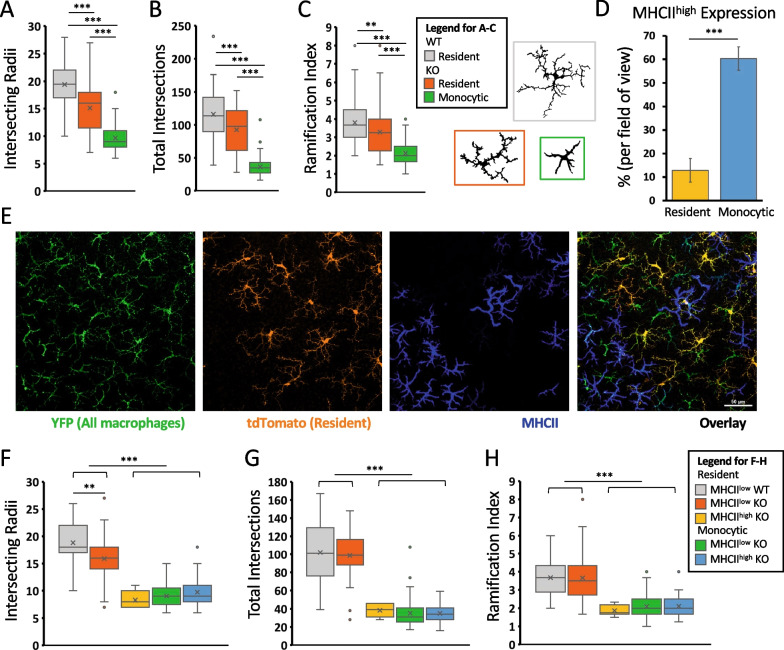
Morphological differences between subtypes of retinal macrophages. **A**–**C** Sholl analysis of resident (YFP^+^tdTomato^+^) and monocytic (YFP^+^tdTomato^−^) macrophages in WT (*Arr1*^+*/*+^) and KO (*Arr1*^*−/−*^) fate mapping (*Cx3cr1*^+*/YFP−CreER*^* Ai9*^*tdTomato*^ post-tamoxifen) flatmount retinas. WT resident microglia in the IPL were larger, based on the number of intersecting radii (**A** gray bar), and more complex, based on total intersections and the ramification index (**B**, **C**), than KO resident microglia (orange) or KO monocytic macrophages (green) after 20 days of degeneration. Insets show representative morphologies of WT resident (gray), KO resident (orange), and KO monocytic (green) cells. *n* ≥ 25 cells per group over at least 3 fields of view. **D** After degeneration 11% of all resident macrophages (YFP^+^tdTomato^+^, yellow) and 60% of all monocytic macrophages (YFP^+^tdTomato^−^, blue) express detectable levels of MHCII, with the majority of these MHCII^high^ cells being monocyte-derived (mean ± SE; *n* = 8 fields of view/z-stack, 341 cells in total). **E** Representative maximum intensity projection through the IPL of a KO fate mapping retina after 20 days of light, labeled for all macrophages (YFP, green), resident macrophages (tdTomato, orange), and MHCII (blue). Cells with detectable levels of MHCII immunostaining were categorized as MHCII^high^ while those with undetectable levels were categorized as MHCII^low^ since we could not be sure based solely on IHC that cells were MHCII negative. **F**–**H** Sholl analysis revealed that KO MHCII^low^ resident microglia morphologically resemble WT resident microglia while MHCII^high^ resident microglia are morphologically similar to monocytic macrophages based on the number of intersecting radii (**F**), total number of intersections (**G**), and ramification index (**H**). *n* ≥ 3 fields of view / z-stacks per genotype, resulting in 7–55 cells per group. **p < 0.01, ***p < 0.001

To further compare the two lineages of macrophages, we quantified the morphologies of these cells in histological flatmounts using Sholl analysis. Resident microglia (YFP^+^tdTomato^+^) in control (WT, *Arr1*^+*/*+^, 20 days light) and degenerated (KO, *Arr1*^*−/−*^, 20 days light) retinas, as well as monocyte-derived macrophages (YFP^+^tdTomato^−^) in degenerated retinas were examined. Consistently, the resident microglia were larger and more complex than the peripherally derived macrophages. On average, microglia covered a larger spatial extent than monocyte-derived macrophages, as indicated by intersecting more radii (Fig. 5A), and had more complex morphologies, as indicated by more total intersections and larger ramification index, than monocyte-derived macrophages (Fig. 5B and C, respectively). Interestingly, the microglia after degeneration covered a smaller area (Fig. 5A) and were slightly less complex (Fig. 5B, C) than microglia in healthy retinas. Like the transcriptomic results above, this morphological analysis supports the conclusion that resident microglia after degeneration maintained a slightly activated phenotype.

Because the single-cell sequencing data (Fig. 3) suggested that monocyte-derived macrophages typically express higher levels of MHCII-related genes than microglia, we next examined MHCII expression in retinal flatmounts from the lineage tracing paradigm. As before (Fig. 1B), MHCII expression was not highly expressed in microglia of WT retinas. In contrast, in degenerated KO retinas (20 days of light) 60% of all monocyte-derived (YFP^+^tdTomato^−^) macrophages and 11% of all resident (YFP^+^tdTomato^+^) cells expressed high levels of MHCII (Fig. 5D, E). Sholl analysis revealed that resident cells that were MHCII^low^ had normal morphologies similar to those of WT retinas, and that all monocyte-derived cells and MHCII^high^ resident cells were smaller (Fig. 5F) and less complex (Fig. 5G, H) than normal. In fact, there were no statistical differences between the MHCII^high^ resident cells and monocytic groups. The only statistical difference between the MHCII^low^ resident groups is that after degeneration the cells are slightly smaller on average (Fig. 5F, WT vs KO; ANOVA followed by Tukey’s honest significant difference test, *p* = 0.0038). These results suggest that MHCII staining is a reasonable screening marker for monocyte-derived cells in the retina, although it should not be used as a conclusive identifier because it is likely to also reveal roughly 10% of the resident cells, which could include perivascular macrophages as well as microglia.

### Probing the activation potential of macrophages to a further retinal injury

**Fig. 6:**
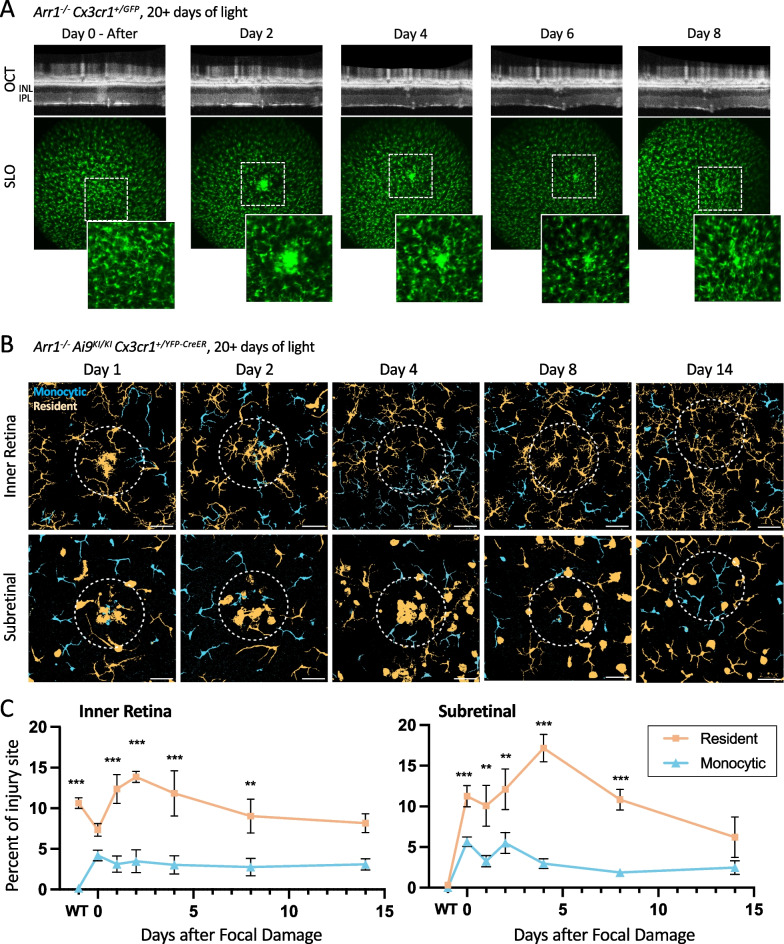
Resident microglia respond to secondary, focal damage following widespread retinal degeneration. **A** Representative in vivo time course of an *Arr1*^*−/−*^* Cx3cr1*^+*/GFP*^ retina after focal laser damage. Retinal macrophages (green, GFP^+^) transiently respond to a focal laser damage administered more than 20 days after onset of light exposure. Days indicate days post-laser injury. Top row: focal damage indicated by increased scattering in OCT. Bottom row: GFP^+^ macrophages initially migrate towards and then away from the focal damage locus. Inset locations indicated by dashed squares. INL = inner nuclear layer; IPL = inner plexiform layer. **B** Representative maximum intensity histological images at 1, 2, 4, 8 and 14 days following focal laser injury administered more than 20 days after onset of light exposure in *Arr1*^*−/−*^* Ai9*^*KI/KI*^* Cx3cr1*^+*/YFP−CreER*^ post-tamoxifen mice in the inner retina (IPL) and corresponding subretinal layers. Images are thresholded, pseudocolored-overlay projections, as in Fig. 4A, where resident cells (predominantly microglia) are displayed in peach and peripherally derived macrophages are indicated in blue. Dashed circle indicates approximate location of the focal damage locus (diameter = 150 μm) and scale bar = 50 μm. See also Additional file 3: Fig. S3, Additional file 4: Fig. S4, Additional file 5: Fig. S5, Additional file 6: Fig. S6, Additional file 7: Fig. S7, Additional file 8: Fig. S8. **C** Significantly more resident cells respond to secondary, focal damage than monocytic cells. Quantification indicates the percent of colored pixels inside the injury area from pseudocolored-overlay images in KO animals after laser damage compared to undamaged KO (0 day) and WT (*Arr1*^+/+^) controls. Mean ± SE; **p < 0.01, ***p < 0.001; *n* = 4–8 laser damage locations in 3–4 retinas (2 mice) per timepoint

Given that the population of retinal CD45^+^ cells are heterogeneous after degeneration and that many cells have a mildly activated transcriptional profile, we next tested the ability of these immune cells to respond to further retinal injury. After photoreceptor degeneration was complete (20 days following light onset), we performed a focal laser injury in *Arr1*^*−/−*^*Cx3CR1*^+*/gfp*^ mice and examined the retinal structure and GFP^+^ immune cells using OCT and SLO, respectively. Similar to the normal microglial response to focal laser damage [19], GFP^+^ cells in the degenerated retina migrated to and clustered at the focal site of damage within a few days. This cluster of cells then gradually disappeared and the GFP^+^ cells again spatially tiled the retina over a period of over a week (Fig. 6A). These results demonstrate that the populations of myeloid cells in the retina after degeneration can respond to further insult.

To determine whether these responding immune cells consist of resident microglia, monocyte-derived macrophages, or both, we performed focal light damage in the fate-mapping (*Arr1*^*−/−*^*Ai9*^*KI/KI*^* Cx3cr1*^+*/YFP−CreER*^) mice after photoreceptor loss was complete and used immunohistochemistry to identify monocytic (YFP^+^tdTomato^−^) and microglial (YFP^+^tdTomato^+^) cells around the focal injury. Both microglia (YFP^+^tdTomato^+^) and monocyte-derived macrophages (YFP^+^tdTomato^−^) were visible in the tight cluster of cells that formed directly at the injury locus in the inner retinal and subretinal layers (Fig. 6B; see also Additional file 3: Fig. S3, Additional file 4: Fig. S4, Additional file 5: Fig. S5, Additional file 6: Fig. S6, Additional file 7: Fig. S7, Additional file 8: Fig. S8), but only the resident microglia showed a significant response to the secondary focal damage. Figure 6C shows that following focal damage (Days 1–4), there was a sharp increase in microglia in both the inner and outer retinal layers at the injury locus, indicating that the neighboring microglia (YFP^+^tdTomato^+^) more readily migrated than the monocyte-derived cells (YFP^+^tdTomato^−^). In the subretinal space, it was particularly noticeable that most of the amoeboid macrophages were resident (YFP^+^tdTomato^+^) in origin. By 14 days, the cluster had resolved and myeloid cells again retiled the retina in both layers. Considering that monocyte-derived macrophages comprise approximately half of the retinal macrophages at the timepoint focal damage was performed (Fig. 4C) and that the majority of macrophages at the damage location were microglial in origin (Fig. 6C), we conclude that monocyte-derived cells residing in the retina are substantially less responsive than resident microglia to further insults.

## Discussion

Blinding diseases like retinitis pigmentosa and macular degeneration arise from loss of photoreceptors over time, concurrent with aging in some disorders. The effect of prior degeneration on the functional states of retinal immune populations could thus influence the susceptibility of the retina to subsequent insults and cell loss over one’s lifespan. Here, we find that photoreceptor degeneration is associated with an increase in the total number of myeloid cells in the retina, and that these cells remain morphologically, transcriptionally, and functionally heterogeneous.

### Dramatic increase in CD45^+^ cells in the retina following degeneration

In previous work, the large increase in myeloid cells in the retina during photoreceptor degeneration in the *Arr1*^*−/−*^ mouse appeared to be the result of both infiltration of monocytes from the periphery and proliferation of myeloid cells within the retina [9]. Here we show that the number of myeloid cells eventually falls as degeneration wanes, but never quite returns to normal pre-degeneration levels. Because loss of photoreceptors during degeneration results in a dramatic decrease in the volume and total number of cells in the retina, even this relatively small increase in total immune cells results in a considerable increase in the density of immune surveillance within the retina. Our laser damage experiments show that this higher density of surveillance does not seem to affect the time course of the microglial response to subsequent injury compared to previous work in otherwise healthy retinas [19]. Whether or not the change in cell number or composition accounts for the apparently poor responsiveness of the monocyte-derived macrophages remains an open question. Interestingly, our EdU experiments show that roughly half of the myeloid cells in the post-degeneration mosaic were born when degeneration was most active (Fig. 1D), but we do not know whether these cells all locally proliferated or to what extent they entered the retina as EdU + newly born monocytes. Our lineage tracing experiments show that roughly half of the immune cells are monocyte-derived (Fig. 4C), but whether or not an equal fraction of microglia and monocyte-derived cells proliferated to re-populate the retina remains a question for future studies.

### Monocyte-derived cells remain in the retina following degeneration

Our flow cytometry data here, in combination with previous work, suggest that these monocyte-derived macrophages arise from monocytes that invaded the retina during the first few days of degeneration, rather than resulting from continued invasion of monocytes throughout the entire course of degeneration. Newly born monocytes express high levels of Ly6C, which then decreases over the cell’s lifetime, especially if the monocyte differentiates into a monocyte-derived macrophage [29]. In previous work, flow cytometry results showed that the number of Ly6C^high^ cells in the *Arr1*^−/−^ retina peaked between 24 and 48 h during the height of photoreceptor degeneration, which corresponded well to the time course of the increase in Müller-cell derived CCL2 expression (monocyte chemoattractant protein) within the retina [10]. In this study, we examined the number of Ly6C^high^ cells in the retina at longer times, after degeneration had slowed and found that the number of Ly6C^high^ returned to non-detectable baseline levels by 10 days (Fig. 2E). Immunohistochemistry and Sholl analysis revealed that these monocyte-derived cells had processes, though were somewhat less ramified than the resident microglia (Fig. 5A–C). Thus although the integrity of the blood retinal barrier has not been explicitly examined in this model, together these results suggest that the monocyte invasion is limited to the peak period of degeneration, and that these monocytes rapidly differentiate into microglia-like macrophages that persist in the retina for at least several more weeks.

The monocyte-derived macrophages that take up residence in the retina after photoreceptor degeneration adopt a ramified morphology that is spatially intermingled with the endogenous microglia (Fig. 4A), similar to the monocyte-derived cells that infiltrate the retina following NaIO_3_ administration to damage the RPE [15]. Unlike the macrophages in other ocular compartments with high turnover rates [37], these monocyte-derived macrophages in the retina are long-lived, and our morphological analysis shows that they are smaller and less complex that resident microglia on average (Fig. 5A–C). The extent of morphological difference between resident and monocyte-derived macrophages may vary by disease. For example, in the NaIO_3_ model of RPE damage, resident and monocyte-derived macrophages were morphologically indistinguishable [15].

Although monocyte-derived macrophages adopt a largely microglial phenotype significant differences in gene expression remain. One such difference readily detectable at the protein level is MHCII. Immunohistochemically, the MHCII^+^ cells observed in the degenerated retina (e.g., Figure 1B) mostly correspond to the monocytic cells (ratio of 6:1 monocyte to microglia-derived cells; Fig. 5D). MHCII^+^ resident macrophages have been observed occasionally in other models of retinal/RPE injury [15, 37]. Notably, here we find that the relatively few resident cells that express high levels of MHCII proteins are morphologically indistinguishable from monocyte-derived macrophages (Fig. 5F–H). Thus, while MHCII staining may be a useful tool for qualitative assessment of peripheral infiltration that is much easier and cheaper than fate-mapping experiments, we emphasize that, like morphology, MHCII expression alone is not sufficient to determine the lineage of a given cell.

Monocyte-derived, or other non-microglial macrophages, have been observed to similarly adopt microglia-like phenotypes and remain in the retina in the absence of continuing or active neuronal loss in previous work. However, those instances have been situations in which there is physical damage [22], disruption of blood–tissue barriers [15, 37], or a pharmacological intervention that ablates the resident microglia [6]. Monocytic cells have been detected in other photoreceptor degenerative disorders, including widespread light damage [20] and age-related macular degeneration [27], although it has not yet been investigated if monocytes permanently engraft into the retina in these instances. To our knowledge, this is the first demonstration of monocyte-derived macrophages taking up long-lived residence in the retina following cell autonomous degeneration. The “new resident’’ monocytic cells adopt a microglia-like morphology and gene enrichment program aligned with a monocyte-derived macrophage phenotype; however, they fail to functionally respond to a subsequent insult as well as the resident microglia. The molecular basis of the apparent functional laxity of the monocyte-derived cells remains to be determined.

In the *Arr1*^*−/−*^ experiments presented here, monocyte-derived macrophages adopted a microglia-like phenotype over the course of days, consistent with evidence from other studies that the retinal environment promotes a microglia-like phenotype for myeloid cells. For example, after pharmaceutical ablation of retinal microglia, non-microglial immune cells from the periphery invade and adopt morphologies and gene expression reminiscent of microglia [6, 17, 22]. Non-microglial repopulation following microglia ablation has not been observed in the brain thus far [7]. However, following whole body irradiation and bone marrow or hematopoietic stem cell transplantation, non-microglial macrophages can invade the brain and develop similar microglia-like morphologies and some shared gene expression [28]. This study identified a non-microglia-specific gene expression program expressed following LPS challenge [28], and some of these genes were found to be similarly expressed in the small cluster of inflammatory macrophages identified here (Cluster #4), but are notably absent in the more microglia-like cells (Cluster #3, Fig. 3C). All together the CNS seems to promote a microglia-like phenotype for mononuclear phagocytes, although it remains unclear to what extent this phenomenon may differ between the brain and retina.

A specific disease-associated microglia (DAM) phenotype first observed at the single-cell level in a mouse model of Alzheimer’s disease [11] has been observed in a wide range of neurodegenerative disorders [4] and similar changes in microglial gene expression have been observed in several models of retinal degeneration [21, 24, 37]. Many DAM-associated genes were indeed expressed by activated microglia in this study, and interestingly these genes were also expressed in the microglia-like macrophages cluster (Fig. 3C). This phenotype of activation was not observed in the small cluster of pro-inflammatory monocyte-derived macrophages, indicating that this is not a pan-activation phenotype in this model.

Unexpectedly, many of the most highly expressed genes in the monocyte-derived macrophage cluster in our single-cell dataset have recently been identified as key genes expressed by various macrophage subsets in peripheral CNS tissues, such as the meninges and choroid plexus [35]. However, to what extent these conserved gene expression programs are indicative of shared functions, similar environment, or similar cell lineages remains unknown.

### Heterogeneous resolution of neuroinflammation across models of acute degeneration

In the field of retinal degeneration, activation and resolution of the immune response has been investigated in a model of RPE injury that causes photoreceptor loss [15] and following a corneal alkali chemical burn that leads to retinal ganglion cell death [22]. In both models, expression of genes associated with immune activation are detected well after cell loss ceases, suggesting that at least some of the retinal immune cells do not return to rest [15, 22]. Because neither study utilized single-cell transcriptomics, the degree of heterogeneity in those post-degeneration immune populations remains unclear. However, the resolution of the immune response to neuronal loss has been investigated at a single-cell level in a model of facial nerve axotomy [32, 33]. Although this model does not result in widespread neuronal loss like the retinal *Arr1*^*−/−*^ model, the loss of neurons does drive the activation and proliferation of nearby microglia, and the immune response resolves approximately 30 days after injury. In this case, single-cell sequencing revealed that although the majority of microglia return to a resting phenotype, a small number of microglia continue to express many activation-related genes like the retinal degeneration models described above, and similar to the mildly activated microglia phenotype we observe in the single-cell dataset described here (Fig. 3).

Curiously, this persistent, mildly activated phenotype observed in all the above models has similarities to changes observed in microglia during aging. In the aging retina, microglia accumulate in the subretinal space, increase in number, and increase their expression of activation- and inflammation-related genes [3, 14]. Typically, aged microglia are not as responsive to injury as young microglia [14], yet here we find that microglia are still capable of robustly responding to focal injury even after all photoreceptors are lost (Fig. 6B, C). The similarities of microglia in neurodegenerative disorders and those in aging are noticeable and could have implications for further immune challenges or therapeutic interventions with age, especially after potential repeated challenges throughout life.

In summary, we find that photoreceptor degeneration causes long-lasting changes to the retinal immune environment, lasting well past when neuronal loss has completed. Although we find that these retinal macrophages can still respond to further retinal insult, the retinal immune environment does not entirely return to rest, and the microglial and monocytic cells respond differently to subsequent injuries and insults. Determining the precise molecular signals and pathways involved in these distinct subpopulations and their functional long-term differences, if any, will be the focus of future studies. Of particular interest will be determining if this altered immune state contributes to late-stage retinal remodeling, and if these immune cells affect therapeutic interventions such as gene therapies and cell transplants.

## Supplementary Information

**Additional file 1: Figure S1.** Gene enrichment analysis of putative monocyte-derived macrophages. (A) Top enriched gene programs identified in the top 35 most highly differentially expressed genes in the putative monocytic cells (Cluster #3, Fig. 3B) compared to mildly activated microglia (Cluster #2, Fig. 3B). (B) Top enriched gene programs identified in the top 400 most highly expressed genes from putative monocytic cells (Cluster #3, Fig. 3B).

**Additional file 2: Figure S2.** Normalization of flow cytometry cell counts from Fig. 4. Quantification of normalized data for resident (YFP^+^tdTomato^+^) and peripherally derived (YFP^+^tdTomato^−^) macrophages from Fig. 4B-C. (A) Counts of total recorded, alive singlets, and CD45^+^ cells before (0 days), immediately after (7 days), and well after (20 days) photoreceptor loss. (B) Monocytic and resident cells normalized to total recorded cells. The number of recorded cells plummets as the photoreceptors die off during degeneration, causing a large shift in the denominator of the normalized data. (C) Monocytic and resident cells normalized to CD45^+^ cells. The number of CD45^+^ cells triples during degeneration, causing a shift in the denominator for these data as well. In both sets of normalized data (B and C), there is a statistical difference at Day 0 and Day 7, as in the raw counts shown in Fig. 4C. All graphs show mean ± SE. ** = *p* < 0.01, *** = *p* < 0.001; *n* = 6 retinas (3 mice) per time point.

**Additional file 3: Figure S3.** Method for assigning lineage cell identity. To distinguish between resident and monocytic lineage cells, single channel maximum intensity projections were thresholded and converted into a binary mask, then averaged to identify overlapping pixels (shown in white). Due to incomplete overlap in the histology, cells were manually pseudocolored peach (YFP^+^tdTomato^+^ resident) when two independent reviewers could clearly identify overlap in the cell body and the majority of processes. Nonoverlapping cells from only the YFP channel (shown in gray) were pseudocolored as blue (YFP^+^tdTomato^−^ monocytic). Dashed circle indicates approximate location of the focal damage locus (diameter = 150 μm), and scale bar is 50 μm.

**Additional file 4: Figure S4.** Day 1 response to focal damage following acute photoreceptor degeneration. Two examples of maximum intensity projections through the inner plexiform and the corresponding subretinal layer 1 day after focal laser damage in lineage tracing mice (*Arr1*^*−/−*^* Ai9*^*KI/KI*^* Cx3cr1*^+*/YFP−CreER*^ post-tamoxifen and after 20 days of light exposure). Resident macrophages express both YFP and tdTomato, whereas monocyte-derived macrophages express only YFP. Pseudocolored images have been thresholded and manually pseudocolored peach for YFP^+^tdTomato^+^ resident cells and blue for YFP^+^tdTomato^−^ monocytic cells. Dashed circle indicates approximate location of the focal damage locus (diameter = 150 μm), and scale bar is 50 μm.

**Additional file 5: Figure S5.** Day 2 response to focal damage following acute photoreceptor degeneration. Two examples of maximum intensity projections through the inner plexiform and the corresponding subretinal layer 2 days after focal laser damage in lineage tracing mice (*Arr1*^*−/−*^* Ai9*^*KI/KI*^* Cx3cr1*^+*/YFP−CreER*^ post-tamoxifen and after 20 days of light exposure). In pseudocolored images, YFP^+^tdTomato^+^ resident cells are indicated in peach and YFP^+^tdTomato^−^ monocytic cells in blue. Dashed circle indicates approximate location of the focal damage locus; scale bar is 50 μm.

**Additional file 6: Figure S6.** Day 4 response to focal damage following acute photoreceptor degeneration. Two examples of maximum intensity projections through the inner plexiform and corresponding subretinal layer 4 days after focal laser damage in lineage tracing mice (*Arr1*^*−/−*^* Ai9*^*KI/KI*^* Cx3cr1*^+*/YFP−CreER*^ post-tamoxifen and after 20 days of light exposure). In pseudocolored images, YFP^+^tdTomato^+^ resident cells are indicated in peach and YFP^+^tdTomato^−^ monocytic cells in blue. Dashed circle shows approximate location of the focal damage locus; scale bar is 50 μm.

**Additional file 7: Figure S7.** Day 8 response to focal damage following acute photoreceptor degeneration. Two examples of maximum intensity projections through the inner plexiform and corresponding subretinal layer 8 days after focal laser damage in lineage tracing mice (*Arr1*^*−/−*^* Ai9*^*KI/KI*^* Cx3cr1*^+*/YFP−CreER*^ post-tamoxifen and after 20 days of light exposure). In pseudocolored images, YFP^+^tdTomato^+^ resident cells are shown in peach and YFP^+^tdTomato^−^ monocytic cells in blue. Dashed circle indicates approximate location of the focal damage locus; scale bar is 50 μm.

**Additional file 8: Figure S8.** Day 14 response to focal damage following acute photoreceptor degeneration. Two examples of maximum intensity projections through the inner plexiform and corresponding subretinal layer 14 days after focal laser damage in lineage tracing mice (*Arr1*^*−/−*^* Ai9*^*KI/KI*^* Cx3cr1*^+*/YFP−CreER*^ post-tamoxifen and after 20 days of light exposure). In pseudocolored images, YFP^+^tdTomato^+^ resident cells are indicated in peach and YFP^+^tdTomato^−^ monocytic cells in blue. Dashed circle indicates approximate location of the focal damage locus; scale bar is 50 μm.

## Data Availability

The single-cell dataset generated during the current study is available in the NCBI Gene Expression Omnibus (GEO) repository under accession number GSE211702. The single-cell dataset generated in [24] and analyzed during this study is available in under accession number GSE121081. All other data from this study are available from the corresponding author on reasonable request.

## Abbreviations

BSA: Bovine serum albumin
CNS: Central nervous system
EdU: 5-Ethynyl-2’-deoxyuridine
FACS: Fluorescence-activated cell sorting
FBS: Fetal bovine serum
FC: Flow cytometry
IHC: Immunohistochemistry
INL: Inner nuclear layer
IP: Intraperitoneal
IPL: Inner plexiform layer
KO: Knockout
OCT: Optical coherence tomography
ONL: Outer nuclear layer
PBS: Phosphate buffered saline
PBT: PBS with 0.5% bovine serum albumin and 0.5% Triton X-100
PCA: Principal components analysis
SLO: Scanning laser ophthalmoscopy
WT: Wildtype
