# Supplementary figures and images for: Structural and functional distinctions of co-resident microglia and monocyte-derived macrophages after retinal degeneration
Source: J Neuroinflammation. 2022 Dec 12;19:299. doi: 10.1186/s12974-022-02652-2 (PMC9743742; doi:10.1186/s12974-022-02652-2)

A

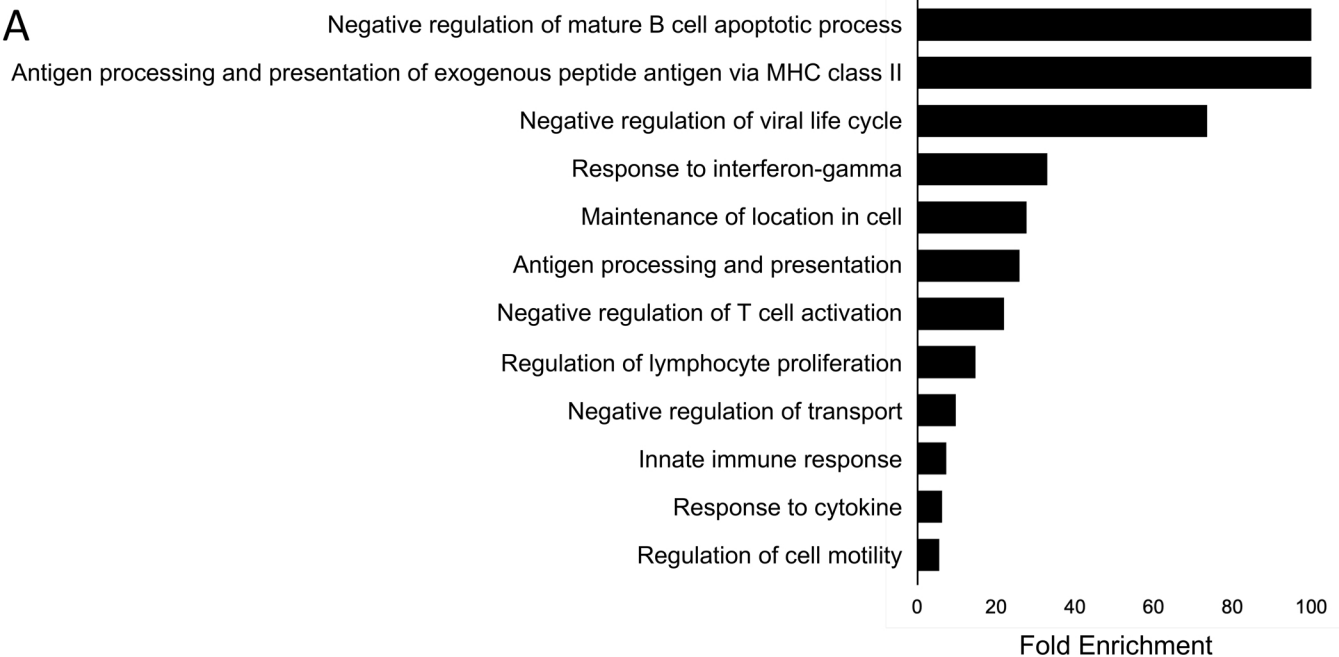

B

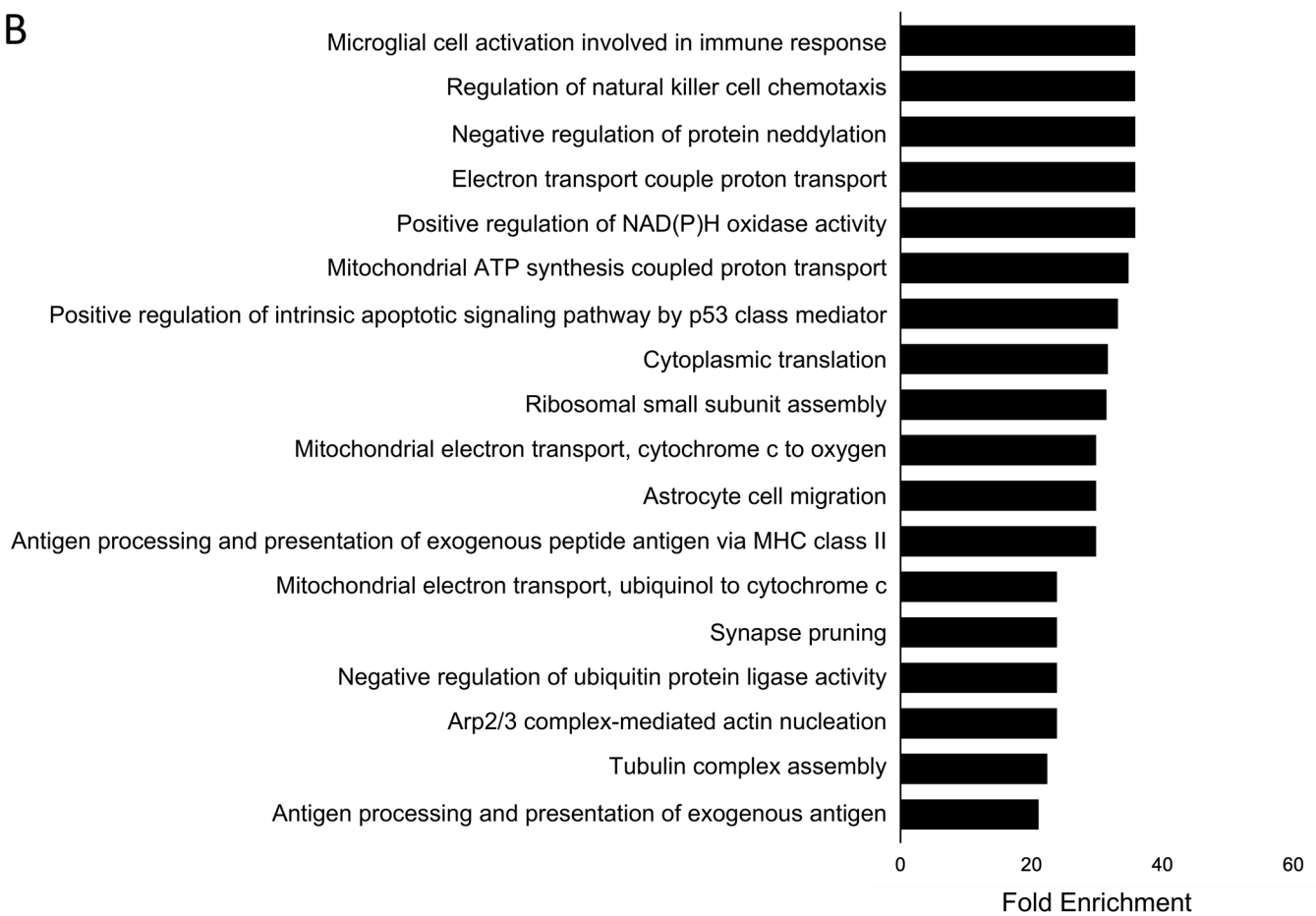

Supplement: Supplementary file 1 — Additional file 1: Figure S1. Gene enrichment analysis of putative monocyte-derived macrophages. (A) Top enriched gene programs identified in the top 35 most highly differentially expressed genes in the putative monocytic cells (Cluster #3, Fig. 3B) compared to mildly activated microglia (Cluster #2, Fig. 3B). (B) Top enriched gene programs identified in the top 400 most highly expressed genes from putative monocytic cells (Cluster #3, Fig. 3B). [file 12974_2022_2652_MOESM1_ESM.pdf]

**A**

Recorded

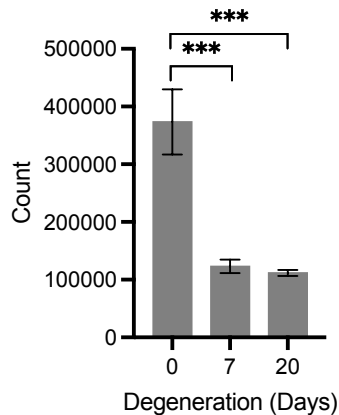

Alive Singlets

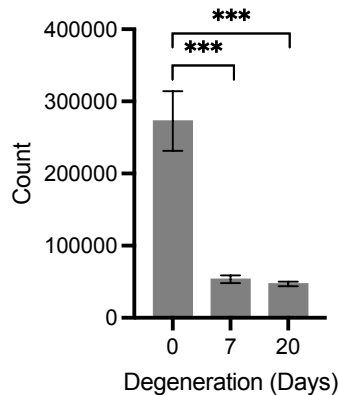

CD45

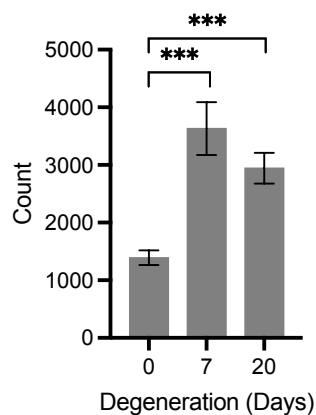**B**

Normalized to Recorded

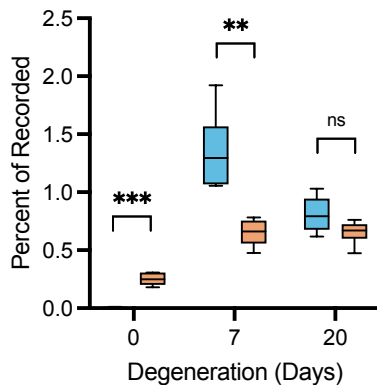**C**Normalized to CD45<sup>+</sup> Population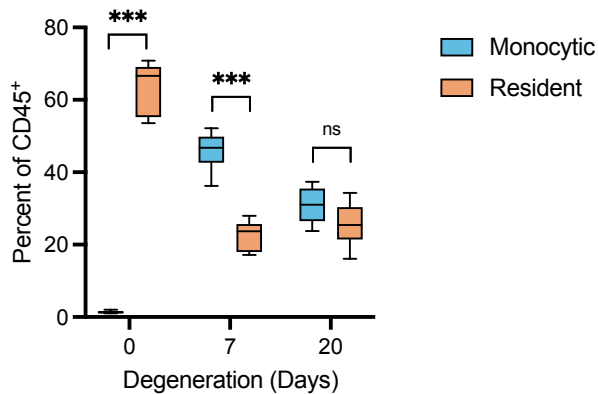

Supplement: Supplementary file 2 — Additional file 2: Figure S2. Normalization of flow cytometry cell counts from Fig. 4. Quantification of normalized data for resident (YFP+tdTomato+) and peripherally derived (YFP+tdTomato−) macrophages from Fig. 4B-C. (A) Counts of total recorded, alive singlets, and CD45+ cells before (0 days), immediately after (7 days), and well after (20 days) photoreceptor loss. (B) Monocytic and resident cells normalized to total recorded cells. The number of recorded cells plummets as the photoreceptors die off during degeneration, causing a large shift in the denominator of the normalized data. (C) Monocytic and resident cells normalized to CD45+ cells. The number of CD45+ cells triples during degeneration, causing a shift in the denominator for these data as well. In both sets of normalized data (B and C), there is a statistical difference at Day 0 and Day 7, as in the raw counts shown in Fig. 4C. All graphs show mean ± SE. ** = p < 0.01, *** = p < 0.001; n = 6 retinas (3 mice) per time point. [file 12974_2022_2652_MOESM2_ESM.pdf]

YFP

tdTomato

IHC

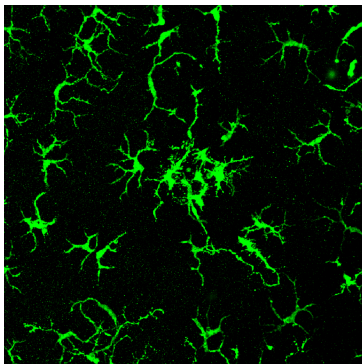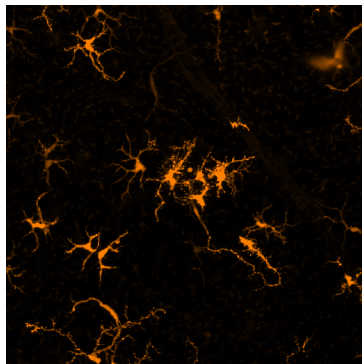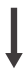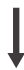

Thresholded

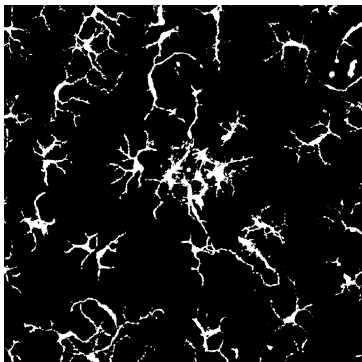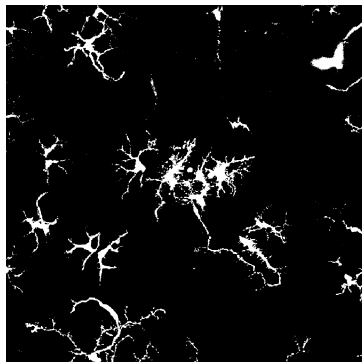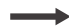

Average value

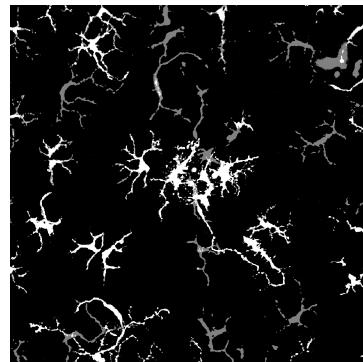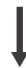

Pseudocolored

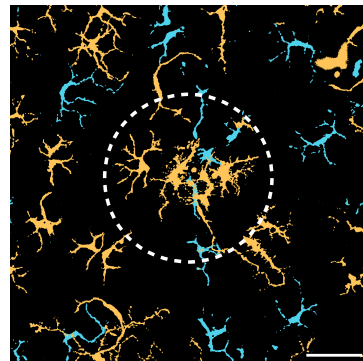

Supplement: Supplementary file 3 — Additional file 3: Figure S3. Method for assigning lineage cell identity. To distinguish between resident and monocytic lineage cells, single channel maximum intensity projections were thresholded and converted into a binary mask, then averaged to identify overlapping pixels (shown in white). Due to incomplete overlap in the histology, cells were manually pseudocolored peach (YFP+tdTomato+ resident) when two independent reviewers could clearly identify overlap in the cell body and the majority of processes. Nonoverlapping cells from only the YFP channel (shown in gray) were pseudocolored as blue (YFP+tdTomato− monocytic). Dashed circle indicates approximate location of the focal damage locus (diameter = 150 μm), and scale bar is 50 μm. [file 12974_2022_2652_MOESM3_ESM.pdf]

Day 1

YFP

tdTomato

Overlay

Pseudocolored

Inner Retina

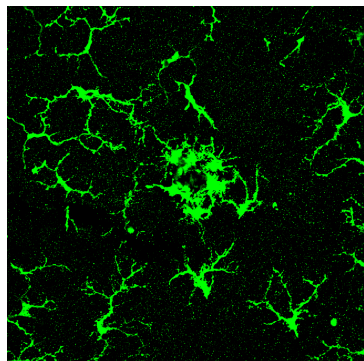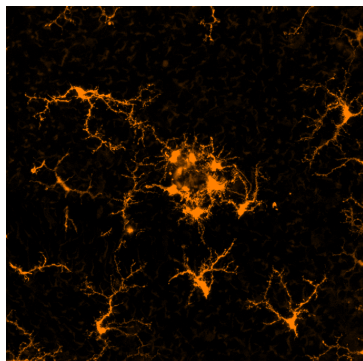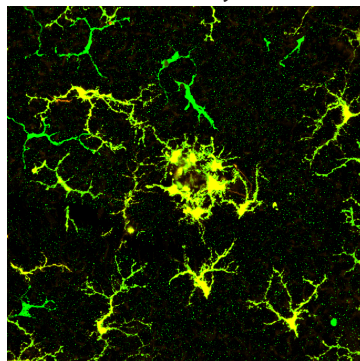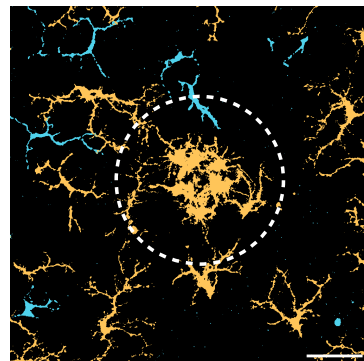

Subretinal

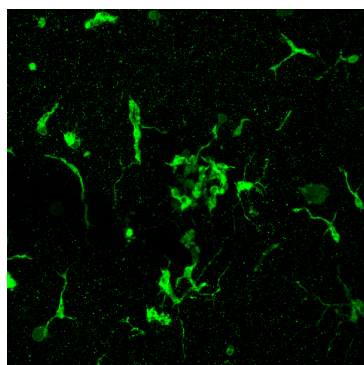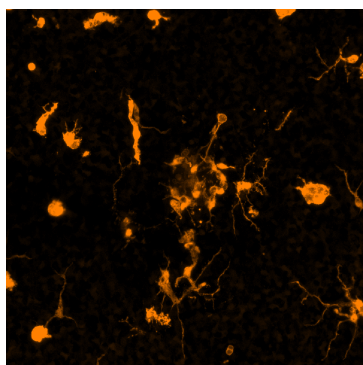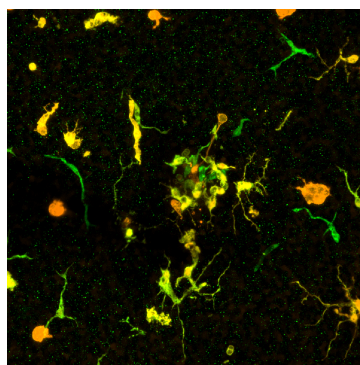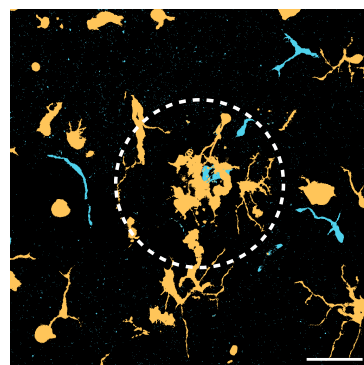

Inner Retina

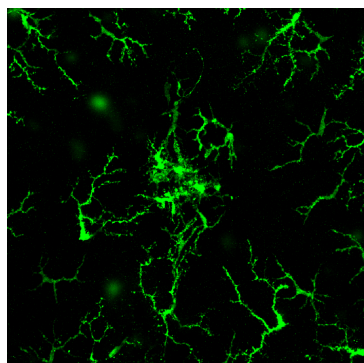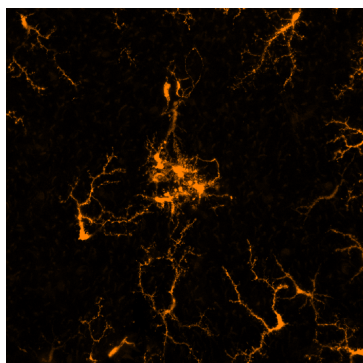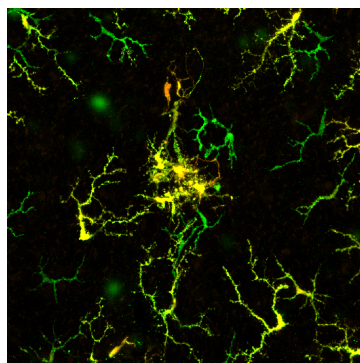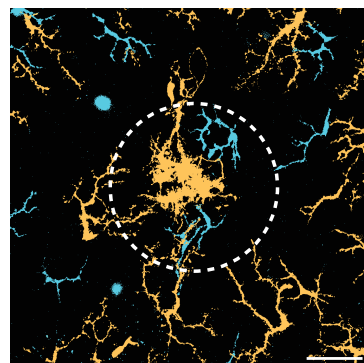

Subretinal

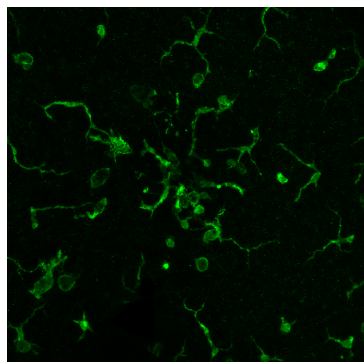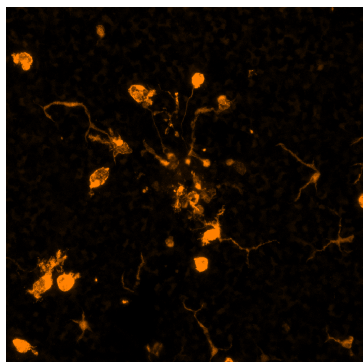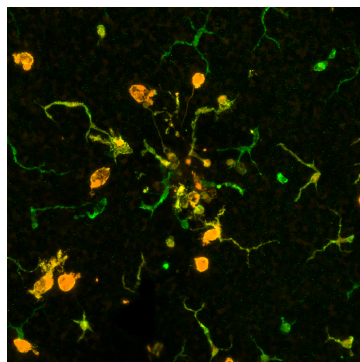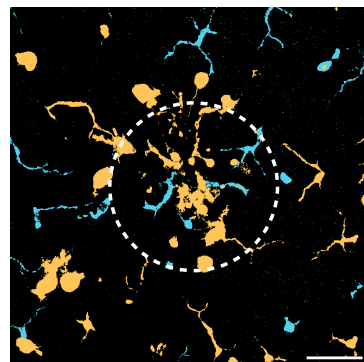

Supplement: Supplementary file 4 — Additional file 4: Figure S4. Day 1 response to focal damage following acute photoreceptor degeneration. Two examples of maximum intensity projections through the inner plexiform and the corresponding subretinal layer 1 day after focal laser damage in lineage tracing mice (Arr1−/− Ai9KI/KI Cx3cr1+/YFP−CreER post-tamoxifen and after 20 days of light exposure). Resident macrophages express both YFP and tdTomato, whereas monocyte-derived macrophages express only YFP. Pseudocolored images have been thresholded and manually pseudocolored peach for YFP+tdTomato+ resident cells and blue for YFP+tdTomato− monocytic cells. Dashed circle indicates approximate location of the focal damage locus (diameter = 150 μm), and scale bar is 50 μm. [file 12974_2022_2652_MOESM4_ESM.pdf]

Day 2

YFP

tdTomato

Overlay

Pseudocolored

Inner Retina

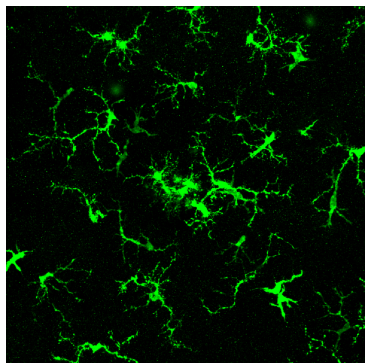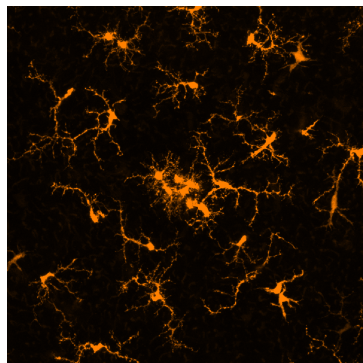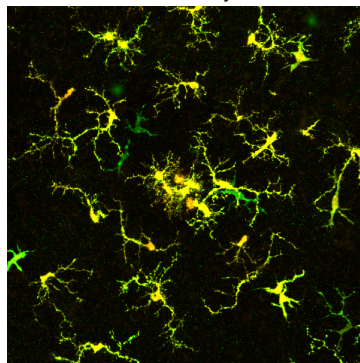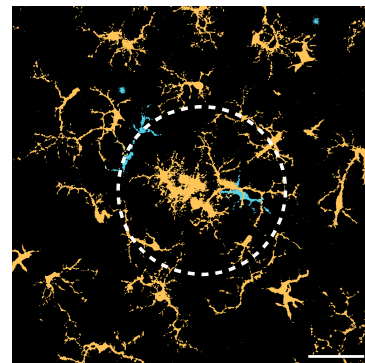

Subretinal

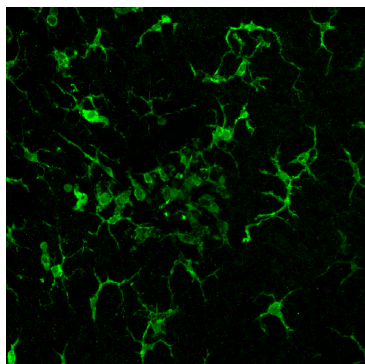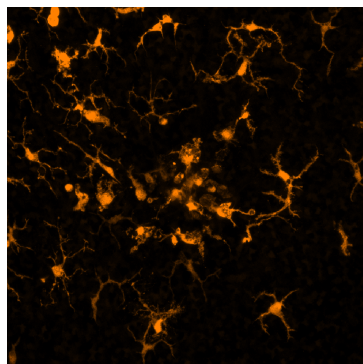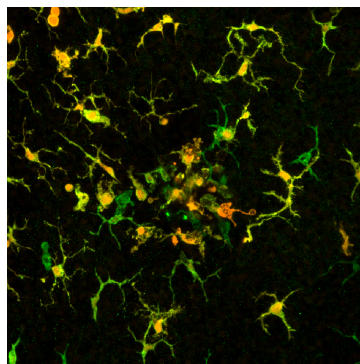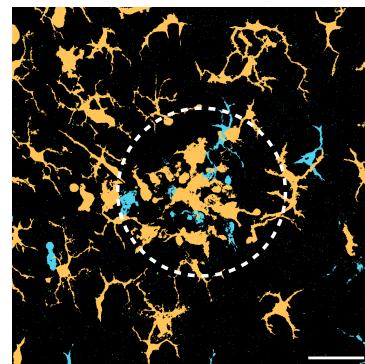

Inner Retina

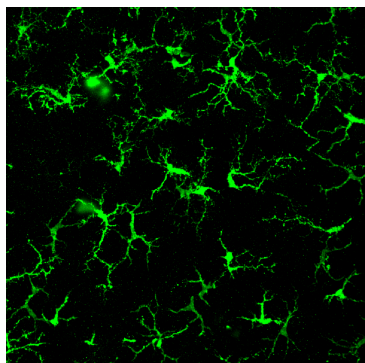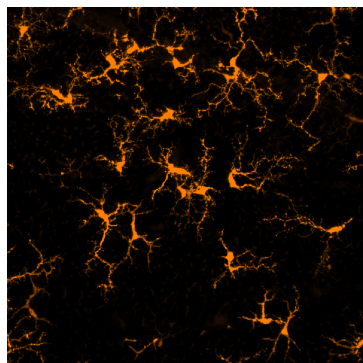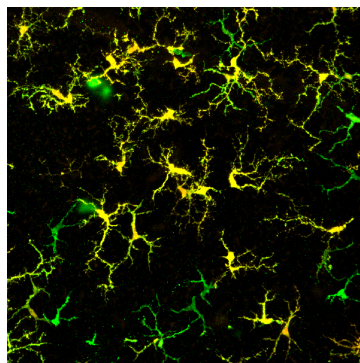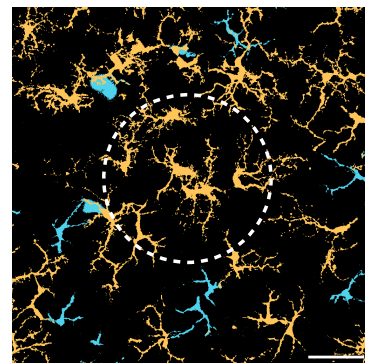

Subretinal

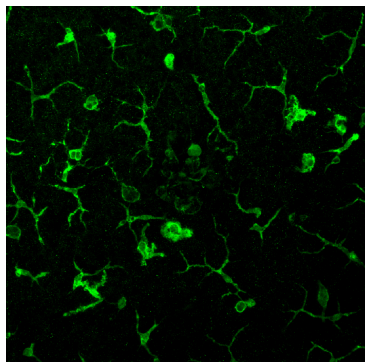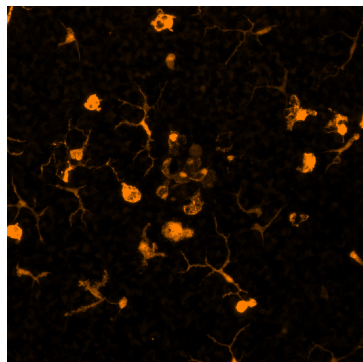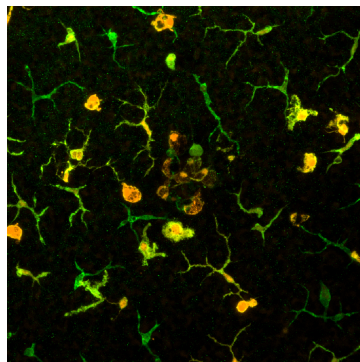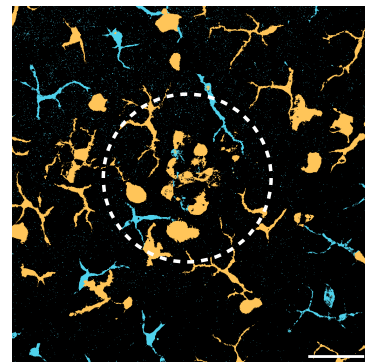

Supplement: Supplementary file 5 — Additional file 5: Figure S5. Day 2 response to focal damage following acute photoreceptor degeneration. Two examples of maximum intensity projections through the inner plexiform and the corresponding subretinal layer 2 days after focal laser damage in lineage tracing mice (Arr1−/− Ai9KI/KI Cx3cr1+/YFP−CreER post-tamoxifen and after 20 days of light exposure). In pseudocolored images, YFP+tdTomato+ resident cells are indicated in peach and YFP+tdTomato− monocytic cells in blue. Dashed circle indicates approximate location of the focal damage locus; scale bar is 50 μm. [file 12974_2022_2652_MOESM5_ESM.pdf]

Day 4

YFP

tdTomato

Overlay

Pseudocolored

Inner Retina

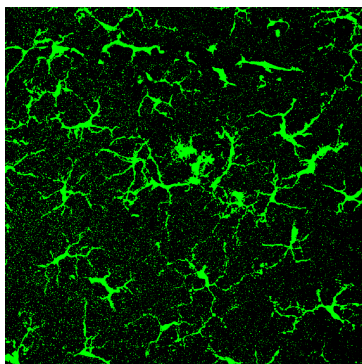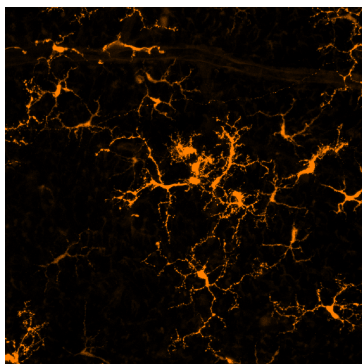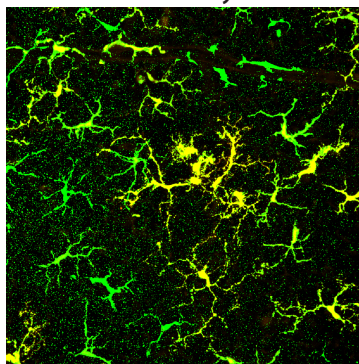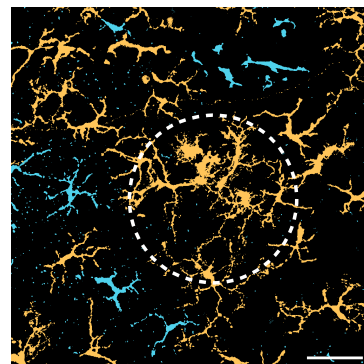

Subretinal

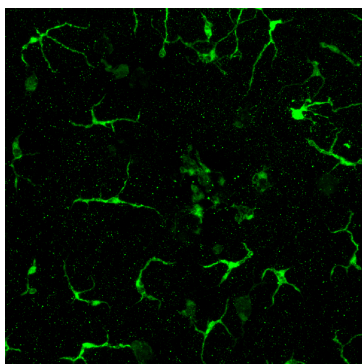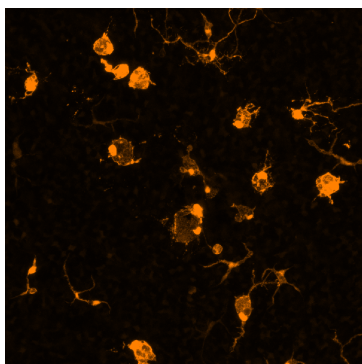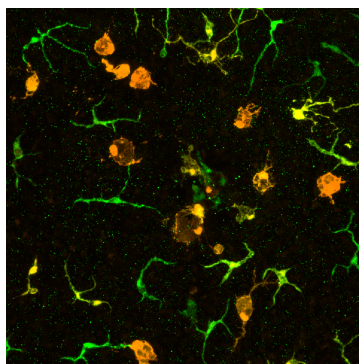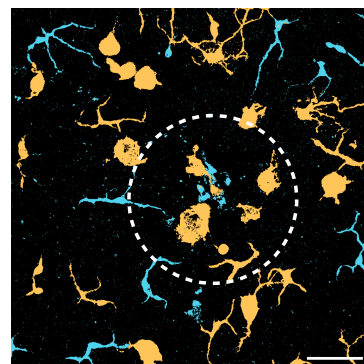

Inner Retina

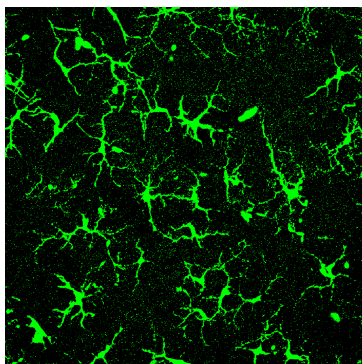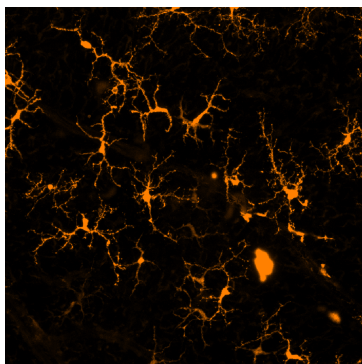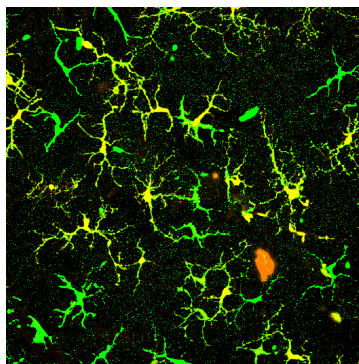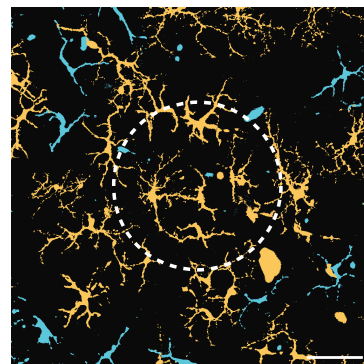

Subretinal

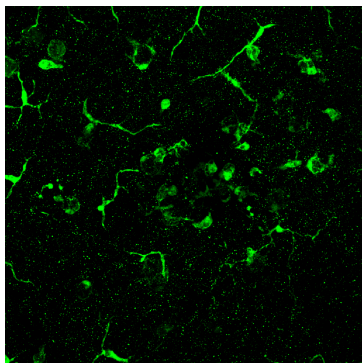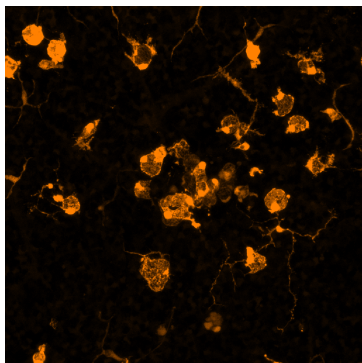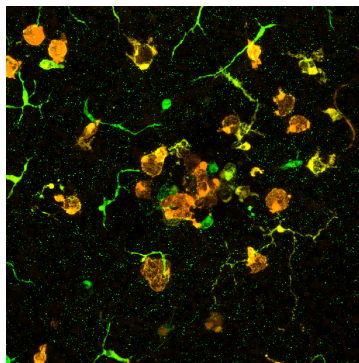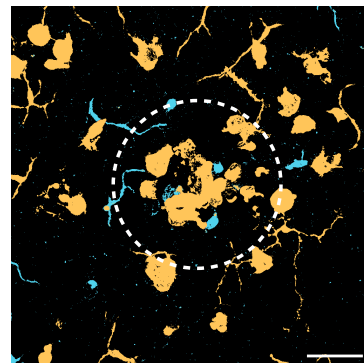

Supplement: Supplementary file 6 — Additional file 6: Figure S6. Day 4 response to focal damage following acute photoreceptor degeneration. Two examples of maximum intensity projections through the inner plexiform and corresponding subretinal layer 4 days after focal laser damage in lineage tracing mice (Arr1−/− Ai9KI/KI Cx3cr1+/YFP−CreER post-tamoxifen and after 20 days of light exposure). In pseudocolored images, YFP+tdTomato+ resident cells are indicated in peach and YFP+tdTomato− monocytic cells in blue. Dashed circle shows approximate location of the focal damage locus; scale bar is 50 μm. [file 12974_2022_2652_MOESM6_ESM.pdf]

Day 8

YFP

tdTomato

Overlay

Pseudocolored

Inner Retina

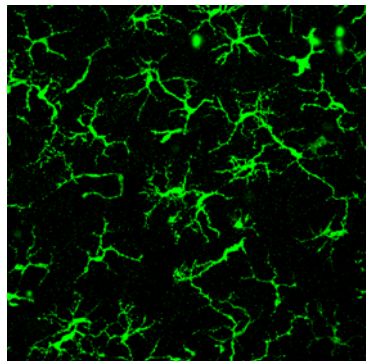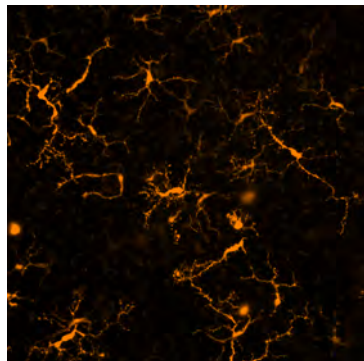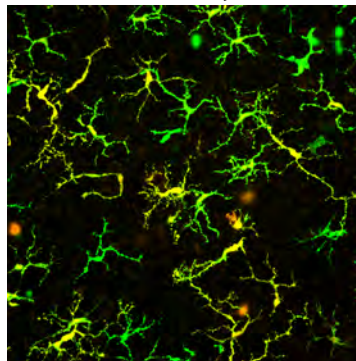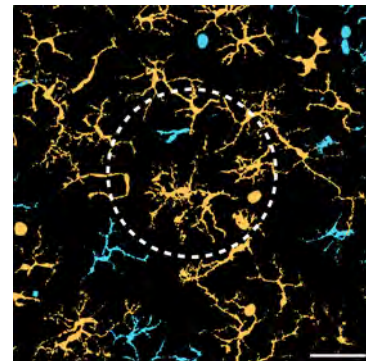

Subretinal

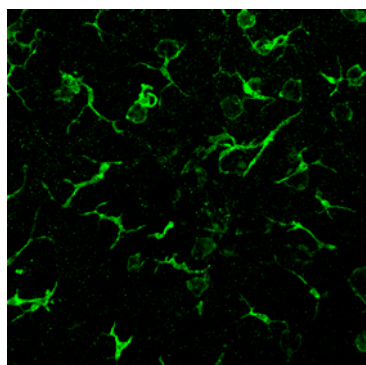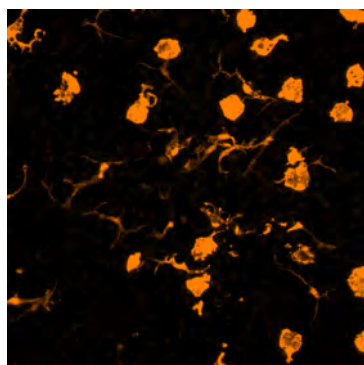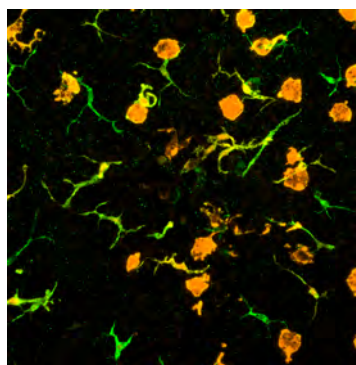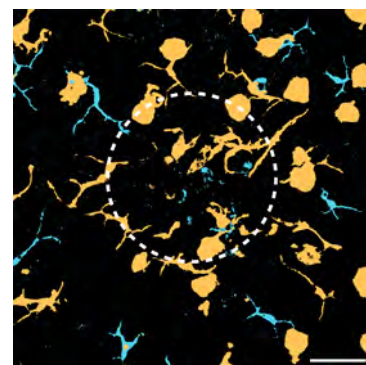

Inner Retina

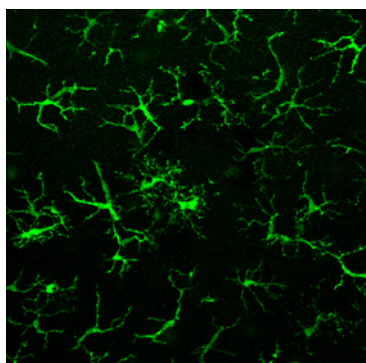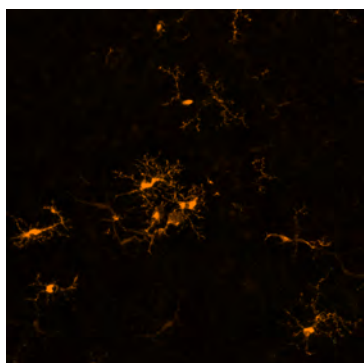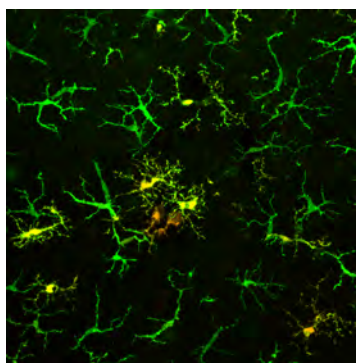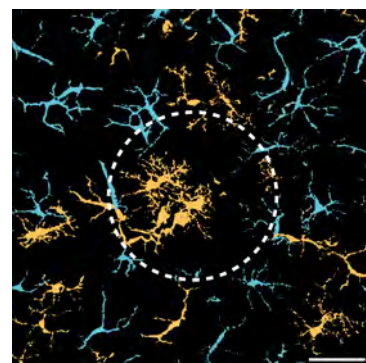

Subretinal

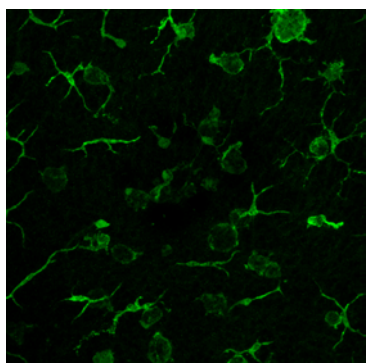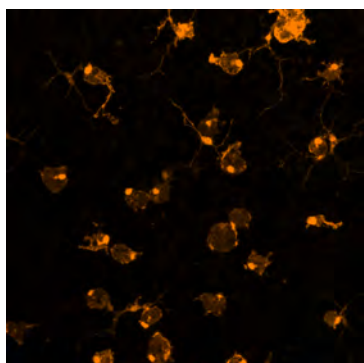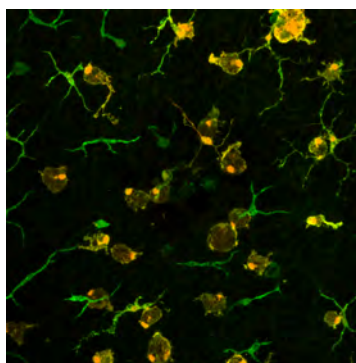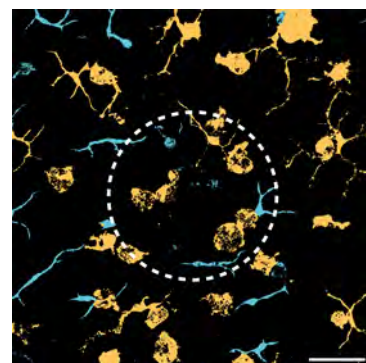

Supplement: Supplementary file 7 — Additional file 7: Figure S7. Day 8 response to focal damage following acute photoreceptor degeneration. Two examples of maximum intensity projections through the inner plexiform and corresponding subretinal layer 8 days after focal laser damage in lineage tracing mice (Arr1−/− Ai9KI/KI Cx3cr1+/YFP−CreER post-tamoxifen and after 20 days of light exposure). In pseudocolored images, YFP+tdTomato+ resident cells are shown in peach and YFP+tdTomato− monocytic cells in blue. Dashed circle indicates approximate location of the focal damage locus; scale bar is 50 μm. [file 12974_2022_2652_MOESM7_ESM.pdf]

Day 14

YFP

tdTomato

Overlay

Pseudocolored

Inner Retina

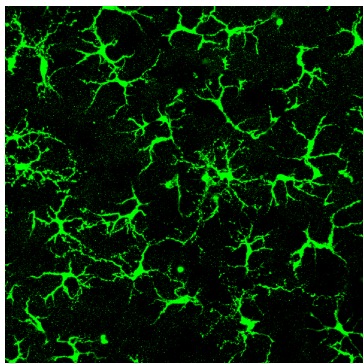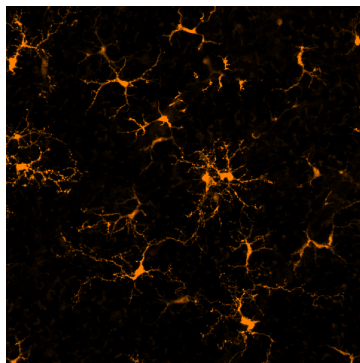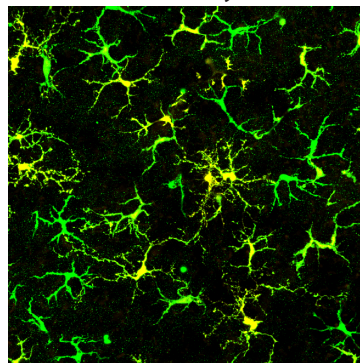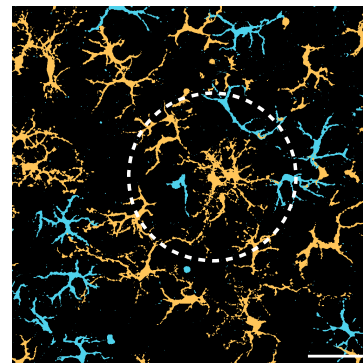

Subretinal

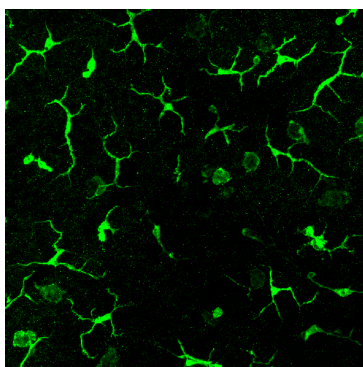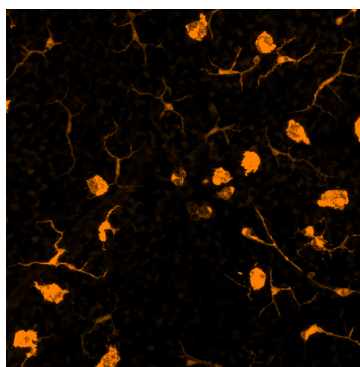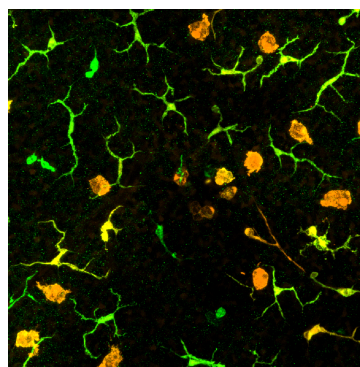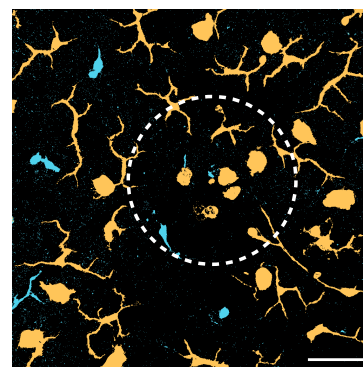

Inner Retina

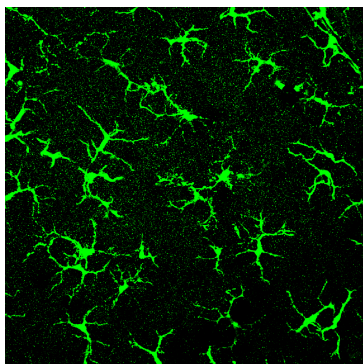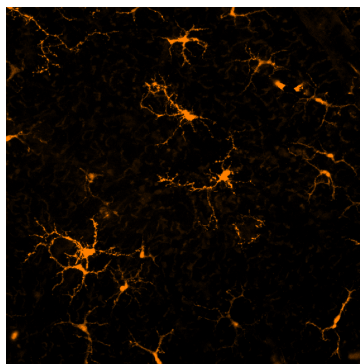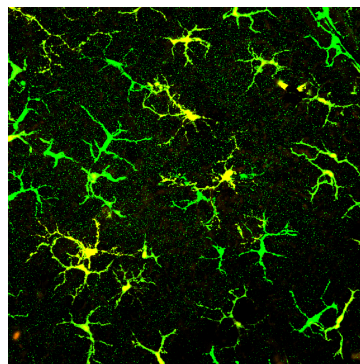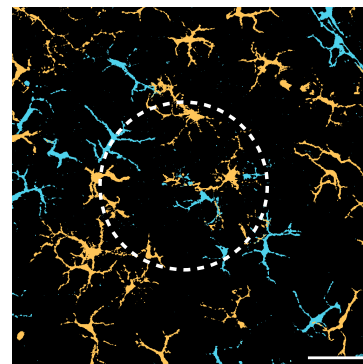

Subretinal

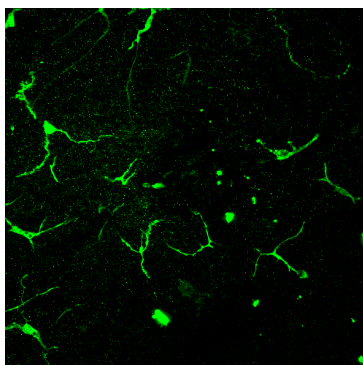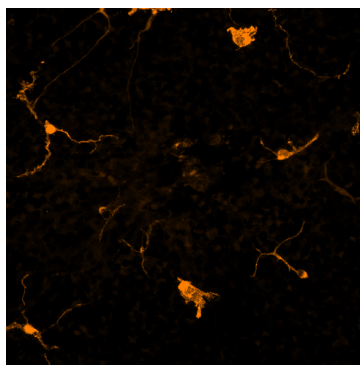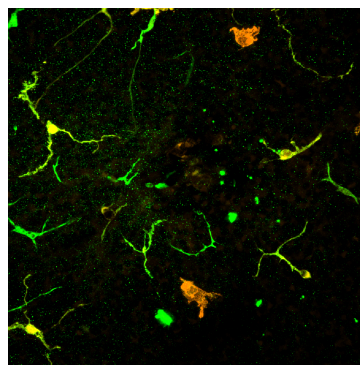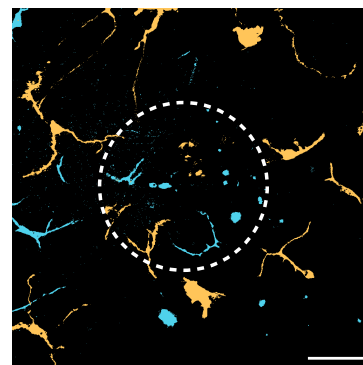

Supplement: Supplementary file 8 — Additional file 8: Figure S8. Day 14 response to focal damage following acute photoreceptor degeneration. Two examples of maximum intensity projections through the inner plexiform and corresponding subretinal layer 14 days after focal laser damage in lineage tracing mice (Arr1−/− Ai9KI/KI Cx3cr1+/YFP−CreER post-tamoxifen and after 20 days of light exposure). In pseudocolored images, YFP+tdTomato+ resident cells are indicated in peach and YFP+tdTomato− monocytic cells in blue. Dashed circle indicates approximate location of the focal damage locus; scale bar is 50 μm. [file 12974_2022_2652_MOESM8_ESM.pdf]
